# Supplementary material for: Cross-sectional analyses of participation in cancer screening and use of hormone replacement therapy and medications in meat eaters and vegetarians: the EPIC-Oxford study
Source: BMJ Open. 2017 Dec 27;7(12):e018245. doi: 10.1136/bmjopen-2017-018245 (PMC5770904; doi:10.1136/bmjopen-2017-018245)
Supplement: Supplementary file 1 [file bmjopen-2017-018245supp001.pdf]

Supplementary text 1: List of 36 named medications on the EPIC-Oxford 10 year follow-up questionnaire.

Alendronate, amlodipine, amitriptyline, aspirin, atenolol, atorvastatin, beclomethasone, bendrofluazide, co-codamol/co-dydramol, contraceptive pill, co-proxamol, diclofenac, digoxin, enalapril, etidronate, frusemide, HRT, ibuprofen, insulin, lisinopril, lithium, Losec/Zoton, metformin, nifedipine, paracetamol, paroxetine, prednisolone, propranolol, Prozac, risedronate, salbutamol, simvastatin, sleeping pills, tamoxifen, thyroxine, warfarin

Supplementary text 2: List of 29 named medical conditions asked on the EPIC-Oxford 10 year follow-up questionnaire.

Cancer (type of cancer), blood clot in leg, blood clot in lung or elsewhere, stroke, transient ischaemic attack, angina, heart attack, palpitations/irregular heart beat (cardiac arrhythmia), diabetes, high blood cholesterol, high blood pressure, asthma, emphysema/chronic bronchitis, thyroid problem, cataract in eye, stomach or duodenal ulcer, bowel polyps, diverticular disease, Crohn’s disease/ulcerative colitis, coeliac disease, osteoporosis, rheumatoid arthritis, osteoarthritis, depression/anxiety, gallstones, gallbladder removed, epilepsy, multiple sclerosis, enlarged prostate (men only)

Supplementary table 1: Sensitivity analyses using data from the 5 year follow-up questionnaire or with further adjustment for possible confounders.

	Diet group, prevalence ratio (95% CI)			
**Sensitivity analyses / Health behaviour of interest**	**Meat eaters**	**Fish eaters**	**Vegetarians**	**Vegans**
**Using data from the 5 year follow-up questionnaire**				
Breast screening[1]	1.00 (ref)	0.95 (0.90-1.00)	0.94 (0.89-0.99)	0.77 (0.67-0.89)
Hormone replacement therapy use[1]	1.00 (ref)	0.82 (0.76-0.89)	0.73 (0.67-0.80)	0.52 (0.40-0.68)
**Further adjustment for confounders**				
Breast screening[2]	1.00 (ref)	0.97 (0.92-1.02)	0.94 (0.89-0.99)	0.83 (0.71-0.96)
Cervical screening[2]	1.00 (ref)	1.00 (0.97-1.03)	0.99 (0.96-1.02)	0.95 (0.99-1.02)
Prostate specific antigen testing[2]	1.00 (ref)	1.00 (0.85-1.17)	0.83 (0.72-0.97)	0.76 (0.53-1.08)
Hormone replacement therapy use[2]	1.00 (ref)	0.81 (0.73-0.88)	0.76 (0.69-0.84)	0.44 (0.31-0.63)
Any medication use[3]	1.00 (ref)	0.92 (0.88-0.97)	0.94 (0.90-0.98)	0.71 (0.63-0.81)

1. Adjusted for age at follow-up (<40, 40-44, 45-49, 50-54, 55-59, 60-64, 65-69, 70-74, ≥75 years, as appropriate according to the age range of included participants), region of residence (eight regions), and self-reported current health (excellent, good, fair, poor, unknown).
2. Adjusted for age at follow-up (<40, 40-44, 45-49, 50-54, 55-59, 60-64, 65-69, 70-74, ≥75 years, as appropriate according to the age range of included participants), region of residence (eight regions), self-reported current health (excellent, good, fair, poor, unknown), smoking status (never, former, current, unknown), alcohol consumption (<1 g/day, 1-7 g/day, 8-15 g/day, ≥16 g/day), Townsend index of area-level deprivation (quartiles and unknown), and education level (no qualifications, basic secondary e.g. O level, higher secondary e.g. A level, degree, unknown).
3. Adjusted for the cross-classification of sex and age at follow-up (<40, 40-44, 45-49, 50-54, 55-59, 60-64, 65-69, 70-74, ≥75 years), region of residence (eight regions), self-reported current health (excellent, good, fair, poor, unknown), number of self-reported illnesses or conditions (0, 1, 2, 3, ≥4), smoking status (never, former, current, unknown), alcohol consumption (<1 g/day, 1-7 g/day, 8-15 g/day, ≥16 g/day), Townsend index of area-level deprivation (quartiles and unknown), and education level (no qualifications, basic secondary e.g. O level, higher secondary e.g. A level, degree, unknown).
