# Supplementary material for: Cross-sectional analyses of participation in cancer screening and use of hormone replacement therapy and medications in meat eaters and vegetarians: the EPIC-Oxford study
Source: BMJ Open. 2017 Dec 27;7(12):e018245. doi: 10.1136/bmjopen-2017-018245 (PMC5770904; doi:10.1136/bmjopen-2017-018245)
Supplement: Author's manuscript [file bmjopen-2017-018245.draft_revisions.pdf]

BMJ Open is committed to open peer review. As part of this commitment we make the peer review history of every article we publish publicly available.

When an article is published we post the peer reviewers' comments and the authors' responses online. We also post the versions of the paper that were used during peer review. These are the versions that the peer review comments apply to.

The versions of the paper that follow are the versions that were submitted during the peer review process. They are not the versions of record or the final published versions. They should not be cited or distributed as the published version of this manuscript.

BMJ Open is an open access journal and the full, final, typeset and author-corrected version of record of the manuscript is available on our site with no access controls, subscription charges or pay-per-view fees (http://bmjopen.bmj.com).

If you have any questions on BMJ Open's open peer review process please email editorial.bmjopen@bmj.com

BMJ Open

Cross-sectional analyses of participation in cancer screening and use of hormone replacement therapy and medications in meat eaters and vegetarians: the EPIC-Oxford study.

Journal:	*BMJ Open*
Manuscript ID	bmjopen-2017-018245
Article Type:	Research
Date Submitted by the Author:	15-Jun-2017
Complete List of Authors:	Tong, Tammy Yin Ni; Cancer Epidemiology Unit,<br>Appleby, P; Cancer Epidemiology Unit, University of Oxford<br>Bradbury, Kathryn; Cancer Epidemiology Unit, University of Oxford<br>Key, Timothy; Cancer Epidemiology Unit, University of Oxford
**Primary Subject Heading**:	Epidemiology
Secondary Subject Heading:	Nutrition and metabolism, Oncology
Keywords:	NUTRITION & DIETETICS, ONCOLOGY, HEALTH SERVICES ADMINISTRATION & MANAGEMENT

SCHOLARONE™
Manuscripts

For peer review only - http://bmjopen.bmj.com/site/about/guidelines.xhtml

Cross-sectional analyses of participation in cancer screening and use of hormone replacement therapy and medications in meat eaters and vegetarians: the EPIC-Oxford study.

Tammy Y.N. Tong[1], Paul N. Appleby[1], Kathryn E. Bradbury[1], Timothy J. Key[1]

1. Cancer Epidemiology Unit, Nuffield Department of Population Health, University of Oxford, UK

Corresponding author: Tammy Y.N. Tong

Postal address: Cancer Epidemiology Unit, University of Oxford, Richard Doll Building, Old Road Campus, Oxford, OX3 7LF

Tel: +44 (0) 1865 289653

Email: tammy.tong@ceu.ox.ac.uk

Word count: 2996 words

Keywords: vegetarians; cancer screening; hormone replacement therapy; medication use

For peer review only - http://bmjopen.bmj.com/site/about/guidelines.xhtml

ABSTRACT (250 words)

Objectives: To examine differences in health-related behaviours such as screening or testing for cancer, use of hormone replacement therapy (HRT), and use of other medications in different diet groups.

Design: We studied 31,260 participants across four diet groups (18,155 meat eaters, 5,012 fish eaters, 7,179 vegetarians, 914 vegans) in the UK EPIC-Oxford cohort. Questions were asked in follow-up questionnaires regarding participation in breast screening, cervical screening, prostate specific antigen (PSA) testing, use of HRT, and use of medications for the past four weeks. Using Poisson regression, we estimated the prevalence ratios (PR) for each outcome across people of different diet groups, using meat-eaters as the reference group.

Results: Compared to meat-eaters, vegetarian (PR; 95% confidence interval: 0.94; 0.89, 0.98) and vegan (0.82; 0.71, 0.95) women had lower participation in breast screening, and vegetarian men were less likely to undergo PSA testing (0.82; 0.71, 0.96). No differences were observed among women for cervical screening. In women, all non-meat eating groups had lower use of HRT compared to meat-eaters (*p* heterogeneity$<$0.0001). Lower reported use of any medication was observed for participants in all non-meat eating groups with no or one self-reported illness (*p* heterogeneity$\leq$0.0002). No heterogeneity was observed across the diet groups for the reported use of specific medication for high blood pressure, high blood cholesterol, asthma, diabetes, and thyroid disease.

Conclusions: Differences in breast screening, PSA testing, HRT use and overall medication use were observed across the diet groups. Whether such differences contribute to differential long-term disease risks requires further study.

For peer review only - http://bmjopen.bmj.com/site/about/guidelines.xhtml

Strengths and limitations of this study

- This study is the first to simultaneously examine prevalence of breast and cervical cancer screening, prostate specific antigen testing, hormone replacement therapy (HRT) use and medication use in different diet groups.
- The study includes a large number of participants recruited from across different regions in the United Kingdom, with a high proportion of fish eaters, vegetarians, and vegans.
- Recall bias is possible because assessment of cancer screening or testing, HRT use and medication use was based on self-report, although there is no indication that such misclassification bias should differ by diet group.
- The study is cross-sectional and we cannot infer causality.

For peer review only - http://bmjopen.bmj.com/site/about/guidelines.xhtml

INTRODUCTION

People of different habitual diet groups have been shown to have different health characteristics. Compared to meat eaters, vegetarians generally have lower BMI, blood pressure, and circulating low density lipoprotein cholesterol levels [1–3], characteristics likely to reduce disease risk. However, evidence on the long-term risk of many non-communicable diseases across people of different diet groups remains inconclusive. For example, although both a United Kingdom (UK) [4] and a United States (US) [5] study reported lower risk of overall cancer incidence with a vegetarian diet, the associations for specific types of cancer are heterogeneous. For cardiovascular diseases, vegetarians in EPIC-Oxford have been observed to have lower ischaemic heart disease risk (hospitalization and death combined) [6], but no significant difference in ischaemic heart disease mortality was observed between diet groups in the same population [7].

The reason for this apparent difference in risk of incident ischaemic heart disease and ischaemic heart disease mortality in vegetarians is unclear. One possible explanation could be the differential use of appropriate medications in the different diet groups, which subsequently influence disease mortality. In a Belgian population for example, vegetarians had a lower use of prescription medications compared to non-vegetarians, but similar use of non-prescription drugs [8]. On the other hand, differences in other health related behaviours, such as participation in cancer screening or use of hormone replacement therapy (HRT), may also contribute to differences in cancer risk across the diet groups. Results from a Swedish cohort [9] and a US cohort [10] showed that vegetarians (including vegans and people who ate fish but not meat) had lower odds of attending breast screening and prostate cancer screening respectively, when compared to meat eaters. Overall, literature on participation in screening and use of medication across people of different diet groups is scarce, and to our knowledge no studies have specifically examined the use of HRT in different diet groups.

For peer review only - http://bmjopen.bmj.com/site/about/guidelines.xhtml

Therefore, the aim of this study was to assess participation in cancer screening or testing, and use of HRT and other medications among people of different diet groups in a large population-based cohort in the UK with a high percentage of vegetarians.

METHODS

Study population

The EPIC-Oxford study is a UK based cohort recruited between 1993 and 1999. The study protocol was approved by a Multicentre Research Ethics Committee (Scotland A Research Ethics Committee) and participants gave written informed consent. Details of the recruitment process have been described previously [1]. In brief, a combination of general practitioner (GP) recruitment and postal recruitment was used. The GP recruitment invited men and women aged 35 to 59 years registered with participating GPs and recruited 7,421 participants. The postal recruitment was targeted at vegetarians, vegans, and other people interested in diet and health, and recruited 57,990 participants aged 20 or above. All participants included in this analysis completed a full recruitment questionnaire which asked about their habitual diet and other health and lifestyle characteristics. A follow-up questionnaire was then sent to surviving participants approximately 5 years after recruitment (mostly from 2000 to 2003), and a second follow-up questionnaire was mailed approximately 10 years after recruitment (mostly in 2007). In the follow-up questionnaires, updated information was gathered on diet, health and lifestyle, including self-reported current health.

For peer review only - http://bmjopen.bmj.com/site/about/guidelines.xhtml

Assessment of diet group

In the recruitment questionnaire and each subsequent follow-up questionnaire, four questions were asked regarding consumption of meat, fish, dairy products, and eggs. Responses to these questions were used to assign participants to one of four diet groups at each time point: meat eaters (participants who ate meat, irrespective of whether they ate fish, dairy products or eggs); fish eaters (participants who did not eat meat but did eat fish); vegetarians (participants who did not eat meat or fish, but did eat one or both of dairy products and eggs), and vegans (participants who did not eat meat, fish, dairy products, or eggs).

Assessment of participation in screening, HRT and medication use

In the follow-up questionnaires, women were asked if they had ever had a breast screening by mammography, cervical screening by the smear test (only on the 5 year follow-up questionnaire), or used HRT, and men were asked if they had ever had a prostate specific antigen (PSA) test (only on the 10 year follow-up questionnaire). On the 10 year follow-up questionnaire, all participants were asked if they had used any medication for most of the last four weeks, with 36 named medications and a free text field for reporting regular use of any medication not on the list; participants were also asked if they had been diagnosed with any of a list of 29 medical conditions, and the year when the condition was first diagnosed. The corresponding question on medication use on the 5 year questionnaire was shorter, with 20 named medications and 26 medical conditions.

For assessment of specific medication use, five common medical conditions associated with specific medications were identified: high blood pressure (commonly treated with one or more of amlodipine, enalapril, frusemide, propranol, atenolol, bendrofluazide, lisinopril and

For peer review only - http://bmjopen.bmj.com/site/about/guidelines.xhtml

For peer review only

nifedipine), high blood cholesterol (atorvastatin and simvastatin), asthma (beclomethasone and salbutamol), diabetes (insulin and metformin), and thyroid disease (thyroxine).

Statistical analyses

Information on assignment to diet group and assessment of health behaviour from the 10 year follow-up questionnaire was used for our analyses, except for the assessment of participation in cervical screening which was only asked on the 5 year follow-up questionnaire. Participants were excluded from all analysis if they did not answer the relevant questions to be assigned to an appropriate diet group (n=28), or if they did not answer the relevant question on medication use (n=407). For the analyses related to participation in breast screening, cervical screening, PSA testing or HRT use, only women or men who answered the relevant question and were in the specified age group at questionnaire completion were included. The age group specifications were as follows: age 50 to 74 years for breast screening, age 25 to 74 years for cervical screening, age 50 to 84 years for PSA testing, and age 50 to 74 years for HRT use. For HRT use, we further restricted the analysis to post-menopausal women.

For each analysis, we used Poisson regression to estimate prevalence ratios (95% confidence intervals, CI) of cancer screening or testing (breast screening, cervical screening, PSA testing), HRT use, or medication use in different diet groups, using meat eaters as the reference group. The analyses for cancer screening or testing and use of HRT adjusted for age at follow-up (<40, 40-44, 45-49, 50-54, 55-59, 60-64, 65-69, 70-74, ≥75 years as appropriate for the age range included in the analysis), region of recruitment (eight geographical regions across the UK), and self-reported current health (excellent, good, fair, poor, unknown).

For peer review only - http://bmjopen.bmj.com/site/about/guidelines.xhtml

We estimated prevalence ratios of any medication use in each diet group compared to meat eaters, adjusting for the cross-stratification of sex and age at follow-up, region of recruitment, self-reported current health, and the number of self-reported illnesses or conditions (0, 1, 2, 3, ≥4). Additionally, we repeated the analyses stratified by the number of self-reported illnesses or conditions using the above categorisation. Subsequently, for each of high blood pressure, high blood cholesterol, asthma, diabetes, and thyroid disease, we estimated the prevalence ratios of taking appropriate medication by diet group among people diagnosed with each condition in turn, adjusting for covariates as above and additionally for years since reported diagnosis, calculated as year of follow-up questionnaire completion minus reported year of diagnosis (<2, 2-3, 4-5, 6-9, ≥10 years, unknown).

As sensitivity analyses, we repeated the analyses as follows: using data from the 5 year follow-up questionnaire where available; and further adjusting for smoking status, alcohol consumption, and Townsend deprivation index. All statistical analyses were performed using Stata release 14.1 (StataCorp), and P values < 0.05 were considered statistically significant.

RESULTS

Cohort characteristics

After excluding participants who did not answer the relevant questions on diet group or on medication use, data for 31,260 participants who completed the 10 year follow-up questionnaire (18,155 meat eaters, 5,012 fish eaters, 7,179 vegetarians, and 914 vegans) were used for most of the analyses. Characteristics of the participants are presented in **Table 1**. Overall, non-meat eaters were younger, more likely to report having excellent health, less

For peer review only - http://bmjopen.bmj.com/site/about/guidelines.xhtml

likely to be taking medication in the past four weeks, and less likely to have reported any illnesses or conditions.

Participation in screening and use of HRT and medications

Overall, 14,016 women were included in the analyses for breast screening, 27,781 women for cervical screening, and 4,783 men for PSA testing (**Table 2**). In women, compared with meat eaters, vegetarians (prevalence ratio; 95% CI: 0.94; 0.89, 0.98) and vegans (0.82; 0.71, 0.95), but not fish eaters (0.96; 0.92, 1.01) had lower prevalence of breast screening, but no significant heterogeneity was observed between the diet groups for participating in cervical screening (*P*-heterogeneity=0.37). In men, vegetarians (0.82; 0.71, 0.96), but not fish eaters (0.99; 0.85, 1.17) or vegans (0.72; 0.50, 1.02), had significantly lower prevalence of PSA testing compared with meat eaters. For HRT use, women who were non-meat eaters reported lower use (fish eaters: 0.80; 0.73, 0.88; vegetarians: 0.74; 0.68, 0.81; vegans: 0.42; 0.30, 0.60) compared with women who were meat eaters (**Table 3**).

Irrespective of the number of self-reported illnesses and conditions, non-meat eaters reported lower use of any medication (fish eaters: 0.92; 0.87, 0.96; vegetarians: 0.93; 0.89, 0.98; vegans 0.71; 0.63, 0.81) compared with meat eaters (**Table 4**). When the analyses were stratified by the number of self-reported illnesses or conditions, non-meat eaters with no or one illness or condition had reported lower medication use compared to meat eaters (*P*-heterogeneity≤0.0002), but the association was attenuated and no longer statistically significant among participants with two, three, or four or more illnesses or conditions. For medication use specific to several common illnesses and conditions, no significant differences were observed between the diet groups in the reported use of appropriate medications for high blood pressure, high blood cholesterol, asthma, diabetes, or thyroid

For peer review only - http://bmjopen.bmj.com/site/about/guidelines.xhtml

disease, among participants diagnosed with each of these conditions (**Table 5**). Results were consistent when we repeated the analyses where possible using data from the 5 year follow-up questionnaire, or when we further adjusted for smoking, alcohol consumption, and Townsend deprivation index (results not shown).

DISCUSSION

Summary of results

In this UK population based cohort with a large proportion of participants from different diet groups, we generally observed lower participation in breast screening and lower HRT use among women who were non-meat eaters (separately categorised as fish eaters, vegetarians, and vegans) compared with women who were meat eaters. Vegetarian men had lower participation in PSA testing compared with meat eating men, but no significant difference was observed for cervical screening in women across the diet groups. For medication use, non-meat eaters were less likely to report taking medications than meat eaters overall, but there were no significant differences in people reporting two or more illnesses or conditions, or for people reporting taking specific medications for various self-reported conditions.

Comparison with other studies

Few studies have reported on the participation in cancer screening or testing, HRT use or medication use among people of different diet groups, and no study has assessed all these behaviours simultaneously in the same cohort. For breast cancer screening, consistent with our findings, the Swedish Malmö Diet and Cancer Study reported that non-attendance for breast cancer screening was more likely in people who were vegetarians or vegans (odds ratio

For peer review only - http://bmjopen.bmj.com/site/about/guidelines.xhtml

For peer review only

or OR; 95% confidence interval: 1.49; 1.11, 1.99) [9]. Analyses of data from the Adventist Health Study-2 in the United States and Canada showed that all non-meat eaters were less likely to report PSA testing compared with meat eaters (OR 0.50; 0.42, 0.60 for vegans, 0.76; 0.67, 0.86 for vegetarians and 0.79; 0.66, 0.95 for fish eaters) [10], whereas we only observed a lower prevalence among the vegetarians but not the fish eaters (nor the vegans, perhaps because of limited numbers) compared with meat eaters in EPIC-Oxford. However, given the much higher rates of PSA testing in the Adventist Health Study-2 (73.3% versus 31.5% in EPIC-Oxford), attitudes towards screening are likely to be different in the two populations, and therefore the results might not be directly comparable.

For medication use, a cross-sectional study in a Belgian population reported lower use of prescribed medications when comparing vegetarians to a reference Belgian population (25.5% versus 47.3%, $p<0.001$) [8]. While this is consistent with our findings on overall medication use, the study did not assess the use of medications stratified by the number of illnesses, nor did they assess appropriate medication use for specific medical conditions. No studies were found which examined prevalence of cervical screening or HRT use among people of different diet groups. Overall, few studies have examined health related behaviours across habitual diet groups.

Interpretation of findings and implications

Our findings indicate differences in some health related behaviours between people of different diet groups, although the reasons behind such differences are unclear. For the observed differences in screening rates, possible explanations could be related to different attitudes towards the screening programmes. In the UK, all women aged 50 to 70 are invited to attend breast cancer screening clinics [11] and all women aged 25 to 64 are invited for

For peer review only

For peer review only - http://bmjopen.bmj.com/site/about/guidelines.xhtml

cervical screening [12] at regular intervals. On the other hand, there is no national programme for PSA testing, although men over the age of 50 are eligible to arrange for testing via their GP if they wish [13]. In a small Scottish focus group study which asked participants about their attitudes towards cancer screening (n=31 for cervical screening, n=10 for breast screening), the study participants reported that they felt pressure from health care professionals, family and friends to attend cervical screening, and that they also considered it to be normative routine behaviour [14]. On the other hand, they did not report receiving much pressure from health care professionals to attend breast screening, and also said that they felt it was more a matter of personal choice.

Such differences in attitudes towards breast screening and cervical screening are of interest. If such attitudes differed by diet groups, this may help to explain the observed differences in participation for breast screening but not cervical screening, since the latter does not appear to involve so much personal choice. However, relevant evidence is lacking, and both dietary and non-dietary factors which are associated with the participants' decisions to attend either breast screening or PSA testing deserve further study. Moreover, it should also be noted that the GP's attitude towards screening may play a role in influencing the patient's decision to participate even when they are not directly involved with the screening process [15]. The impact of such influences, however, requires further investigation.

Reasons for the observed lower prevalence of HRT use and medication use among people of different diet groups are also unclear. Given that non-meat eaters were more likely to rate their health as good or excellent, one possible explanation is that non-meat eaters were healthier overall and therefore less likely to require any form of treatment including HRT or other medications. However, given the observed differences in medication use among people with no (especially) or only one reported illness or condition, better health among non-meat eaters is unlikely to be the only, or a sufficient explanation for the differences. Non-meat

For peer review only - http://bmjopen.bmj.com/site/about/guidelines.xhtml

eaters may also be reluctant to take medications which are likely to contain animal-derived products [16], or may prefer to use homoeopathic medications [8] or other alternative therapies.

Differential participation in screening for breast or prostate cancer, use of HRT, and use of medications for people of distinct diet groups may ultimately lead to differences in disease incidence or prognosis due to possible detection bias and differential post diagnosis treatment. For example, given the lower rates of breast cancer screening among non-meat eating women, it is possible that the observed incidence of breast cancer in these diet groups underestimates the true incidence owing to detection bias, and that ultimately these women would have a somewhat higher mortality from breast cancer. At the same time, lower prevalence of HRT use among non-meat eating women also deserves attention given the increase in breast cancer risk caused by HRT containing oestrogens and progestogens [17,18]. Further study is warranted to understand why people of different diet groups have differential participation in breast screening or prostate cancer testing, HRT use, and overall medication use, and whether and how these differences are related to future disease risk. Overall, our findings showed some differences in health related behaviours among people of different diet groups, thereby highlighting the need to consider such differences when conducting longitudinal analyses in these populations.

Strengths and limitations

This study is the first to simultaneously examine prevalence of screening, HRT use and medication use in different diet groups. A strength of the study is the large sample size recruited from across different regions in the UK. Additionally, information was collected on a range of factors which may also be associated with the outcomes of interest, allowing

For peer review only - http://bmjopen.bmj.com/site/about/guidelines.xhtml

adjustment for these factors. Of potential limitations, recall bias is possible because assessment of the exposures of interest was based on self-report, although there is no indication that such misclassification bias should differ by diet group. Because of the relatively small number of vegans in our study sample, the role of chance in explaining the findings relating to this diet group cannot be ruled out. As with most population cohorts, some degree of self-selection and healthy cohort bias may also be present.

CONCLUSIONS

In this population, we observed differences in breast screening, PSA testing, HRT use and overall medication use between meat eaters, fish eaters, vegetarians and vegans, but no significant differences between diet groups for cervical screening or medication use in people with two or more illnesses or for specific conditions. The reasons for these differences require further investigation. Nonetheless, such differences may be related to differential observed morbidity or mortality from cancer and other diseases across people of different diet groups, and therefore should be considered in future epidemiological studies.

STATEMENTS

Acknowledgements

We thank all participants in the EPIC-Oxford cohort for their invaluable contribution. The work is supported by the UK Medical Research Council MR/M012190/1 and Cancer Research UK 570/A16491 and C9221/A19170. KEB is supported by the Girdlers' New Zealand Health Research Council Fellowship. TJK is a member of the Vegan Society; the other authors had no conflicts of interest.

For peer review only - http://bmjopen.bmj.com/site/about/guidelines.xhtml

For peer review only

298

299 **Availability of data and materials**

300 Data access policies for EPIC-Oxford are available via the study website (http://www.epic-
301 oxford.org/data-access-sharing-and-collaboration/).

302

303 **Author's contributions**

304 TYNT, PNA, and TJK conceived and designed the research question. TYNT and PNA
305 analysed the data. TYNT wrote the first draft of the manuscript, and PNA, KEB and TJK
306 provided input on data analysis and interpretation of results. All authors revised the
307 manuscript critically for important intellectual content, and read and approved the final
308 manuscript.

For peer review only

For peer review only - http://bmjopen.bmj.com/site/about/guidelines.xhtml

Table 1 Characteristics by diet group of participants in the EPIC-Oxford study who completed the second follow-up questionnaire (n=31260) [1].

Characteristic	Meat eaters	Fish eaters	Vegetarians	Vegans	Total
Number of participants (% female)	18155 (78.2)	5012 (81.8)	7179 (76.3)	914 (66.1)	31260 (78.0)
Mean (SD) age at questionnaire completion, years	58.9 (12.5)	53.8 (12.5)	51.6 (12.7)	50.7 (12.3)	56.1 (13.0)
Smoking status[2], n (%)					
Never smoker	10073 (55.7)	2786 (55.6)	4339 (60.5)	547 (59.9)	17745 (56.9)
Former smoker	6927 (38.3)	1961 (39.2)	2460 (34.3)	330 (36.1)	11678 (37.5)
Current smoker	1094 (6.0)	260 (5.2)	367 (5.1)	36 (3.9)	1757 (5.6)
Mean (SD) alcohol consumption, g/d	8.7 (9.3)	8.2 (8.7)	7.6 (8.9)	6.7 (9.2)	8.3 (9.1)
Self-reported current health[2], n (%)					
Excellent	3713 (21.9)	1323 (28.1)	1950 (28.7)	325 (37.2)	7311 (24.9)
Good	9962 (58.8)	2688 (57.0)	3851 (56.6)	446 (51.0)	16947 (57.8)
Fair	2858 (16.9)	612 (13.0)	876 (12.9)	80 (9.2)	4426 (15.1)
Poor	400 (2.4)	92 (2.0)	122 (1.8)	23 (2.6)	637 (2.2)
Townsend deprivation index[2], n (%)					
Richest category	4463 (27.6)	984 (21.8)	1542 (23.7)	153 (18.3)	7141 (25.5)
Poorest category	3438 (21.2)	1207 (26.8)	1732 (26.7)	285 (34.1)	6662 (23.8)
In same diet group at recruitment, n (%)	15908 (87.7)	3057 (61.1)	6373 (89.1)	573 (62.7)	25911 (83.0)
Taking medication in the past 4 weeks, n (%)	10196 (56.2)	2105 (42.0)	2829 (39.4)	255 (27.9)	15385 (49.2)
Number of reported illnesses and conditions, n (%)					
None	4455 (24.5)	1635 (32.6)	2603 (36.3)	344 (37.6)	9037 (28.9)
One	4724 (26.0)	1472 (29.4)	2170 (30.2)	291 (31.8)	8657 (27.7)
Two	3682 (20.3)	906 (18.1)	1261 (17.6)	154 (16.8)	6003 (19.2)
Three	2404 (13.2)	524 (10.5)	630 (8.8)	74 (8.1)	3632 (11.6)
Four or more	2890 (15.9)	475 (9.5)	515 (7.2)	51 (5.6)	3931 (12.6)
Reported high blood pressure[2], n (%)	4397 (29.2)	686 (16.2)	944 (15.2)	85 (10.6)	6112 (23.2)
and taking appropriate medication, n (%)	2573 (58.5)	357 (52.0)	430 (45.6)	40 (47.1)	3400 (55.6)
Reported high blood cholesterol[2], n (%)	3351 (23.1)	561 (13.5)	645 (10.5)	44 (5.5)	4601 (18.0)
and taking appropriate medication, n (%)	1646 (49.1)	209 (37.3)	243 (37.7)	14 (31.8)	2112 (45.9)
Reported asthma[2], n (%)	1885 (13.6)	496 (12.1)	758 (12.4)	88 (11.1)	3227 (12.9)
and taking appropriate medication, n (%)	737 (39.1)	169 (34.1)	246 (32.5)	17 (19.3)	1169 (36.2)
Reported diabetes[2], n (%)	707 (5.2)	75 (1.9)	119 (2.0)	7 (0.9)	908 (3.7)
and taking appropriate medication, n (%)	446 (63.1)	41 (54.7)	84 (70.6)	6 (85.7)	577 (63.5)
Reported thyroid disease[2], n (%)	1545 (11.1)	380 (9.2)	465 (7.6)	56 (7.1)	2446 (9.8)
and taking appropriate medication, n (%)	1191 (77.1)	273 (71.8)	337 (72.5)	37 (66.1)	1838 (75.1)

1. Based on participant characteristics at the time of the second follow-up questionnaire (completed approximately10 years from baseline, around 2007).
2. Unknown for some participants.

For peer review only

For peer review only - http://bmjopen.bmj.com/site/about/guidelines.xhtml

Table 2 Participation in screening by diet group of women and men in the EPIC-Oxford study.

Screening/Diet group	Number answering the relevant question	Number (%) answering in the affirmative	Prevalence ratio (95% CI) [1]
**Breast screening[2]**			
Meat eaters	9239	8813 (95.4)	1.00 (ref)
Fish eaters	2143	1928 (90.0)	0.96 (0.92,1.01)
Vegetarians	2395	2078 (86.8)	0.94 (0.89,0.98)
Vegans	239	182 (76.2)	0.82 (0.71,0.95)
			P-het=0.004
**Cervical screening[3]**			
Meat eaters	15936	15365 (96.4)	1.00 (ref)
Fish eaters	4513	4369 (96.8)	1.00 (0.97,1.03)
Vegetarians	6574	6268 (95.3)	0.98 (0.95,1.01)
Vegans	758	691 (91.2)	0.94 (0.87,1.02)
			P-het=0.37
**Prostate specific antigen testing[4]**			
Meat eaters	3078	1066 (34.6)	1.00 (ref)
Fish eaters	594	181 (30.5)	0.99 (0.85,1.17)
Vegetarians	947	228 (24.1)	0.82 (0.71,0.96)
Vegans	164	33 (20.1)	0.72 (0.50,1.02)
			P-het=0.023

1. All analyses adjusted for age at follow-up (<40, 40-44, 45-49, 50-54, 55-59, 60-64, 65-69, 70-74, ≥75 years, as appropriate according to the age range of included participants), region of residence (eight regions), and self-reported current health (excellent, good, fair, poor, unknown).
2. Included women aged 50 to 74 who answered the relevant question on the second (10 year) follow-up questionnaire.
3. Included women aged 25 to 74 who answered the relevant question on the first (5 year) follow-up questionnaire
4. Included men aged 50 to 84 who answered the relevant question on the second (10 year) follow-up questionnaire.

Table 3 Use of hormone replacement therapy by diet group of women in the EPIC-Oxford study.

Diet group	Number answering the relevant question	Number (%) answering in the affirmative	Prevalence ratio (95% CI) [1]
Meat eaters	6911	3098 (44.8)	1.00 (ref)
Fish eaters	1614	541 (33.5)	0.80 (0.73,0.88)
Vegetarians	1778	541 (30.4)	0.74 (0.68,0.81)
Vegans	188	31 (16.5)	0.42 (0.30,0.60)
			P-het<0.0001

1. Adjusted for age at follow-up 50-54, 55-59, 60-64, 65-69, 70-74 years), region of residence (eight regions), and self-reported current health (excellent, good, fair, poor, unknown). Included post-menopausal women aged 50 to 74 who answered the relevant question on the second (10 year) follow-up questionnaire.

For peer review only - http://bmjopen.bmj.com/site/about/guidelines.xhtml

Table 4 Medication use by number of self-reported illnesses or conditions and diet group of participants in the EPIC-Oxford study. [1]

Number of self-reported illnesses or conditions / Diet group	Number of participants	Percentage taking any medication	Prevalence ratio (95% CI)[2]
**Any number[3]**			
Meat eaters	18155	56.2	1.00 (ref)
Fish eaters	5012	42.0	0.92 (0.87-0.96)
Vegetarians	7179	39.4	0.93 (0.89-0.98)
Vegans	914	27.9	0.71 (0.63-0.81)
			P-het<0.0001
**None**			
Meat eaters	4455	16.9	1.00 (ref)
Fish eaters	1635	11.9	0.80 (0.68-0.94)
Vegetarians	2603	11.5	0.80 (0.70-0.92)
Vegans	344	6.1	0.47 (0.30-0.72)
			P-het<0.0001
**One**			
Meat eaters	4724	48.9	1.00 (ref)
Fish eaters	1472	39.1	0.87 (0.80-0.96)
Vegetarians	2170	40.5	0.91 (0.84-0.99)
Vegans	291	29.2	0.69 (0.55-0.85)
			P-het=0.0002
**Two**			
Meat eaters	3682	66.9	1.00 (ref)
Fish eaters	906	58.8	0.94 (0.86-1.04)
Vegetarians	1261	58.1	0.97 (0.89-1.06)
Vegans	154	42.2	0.74 (0.58-0.95)
			P-het=0.082
**Three**			
Meat eaters	2404	82.6	1.00 (ref)
Fish eaters	524	74.0	0.94 (0.84-1.05)
Vegetarians	630	73.0	0.94 (0.84-1.04)
Vegans	74	59.5	0.78 (0.57-1.05)
			P-het=0.22
**Four or more**			
Meat eaters	2890	93.0	1.00 (ref)
Fish eaters	475	86.9	0.96 (0.86-1.06)
Vegetarians	515	88.9	0.98 (0.89-1.09)
Vegans	51	78.4	0.87 (0.63-1.19)
			P-het=0.70

1. Refers to medication use for most of the past four weeks on the second (10 year) follow-up questionnaire, excluding HRT and contraceptive pills.
2. Adjusted for the cross-classification of sex and age at follow-up (<40, 40-44, 45-49, 50-54, 55-59, 60-64, 65-69, 70-74, ≥75 years), region of residence (eight regions), and self-reported current health (excellent, good, fair, poor, unknown).
3. Further adjusted for the number of self-reported illnesses or conditions (0, 1, 2, 3, ≥4).

For peer review only - http://bmjopen.bmj.com/site/about/guidelines.xhtml

For peer review only

Table 5 Medication use for specific conditions by diet group of participants in the EPIC-Oxford study.[1]

Condition/ Diet group	Number reporting the condition (mean years since reported diagnosis)	Number (%) taking appropriate medication	Prevalence ratio (95% CI)[2]
**High blood pressure**[3]			
Meat eaters	4397 (9.8)	2573 (58.5)	1.00 (ref)
Fish eaters	686 (9.3)	357 (52.0)	0.97 (0.86-1.08)
Vegetarians	944 (9.0)	430 (45.6)	0.91 (0.82-1.01)
Vegans	85 (9.0)	40 (47.1)	0.92 (0.67-1.26)
			P-het=0.37
**High blood cholesterol**[4]			
Meat eaters	3351 (6.3)	1646 (49.1)	1.00 (ref)
Fish eaters	561 (5.3)	209 (37.3)	0.88 (0.76-1.01)
Vegetarians	645 (5.5)	243 (37.7)	0.94 (0.81-1.08)
Vegans	44 (7.1)	14 (31.8)	0.74 (0.44-1.26)
			P-het=0.20
**Asthma**[5]			
Meat eaters	1885 (25.3)	737 (39.1)	1.00 (ref)
Fish eaters	496 (23.2)	169 (34.1)	0.98 (0.82-1.17)
Vegetarians	758 (23.4)	246 (32.5)	0.97 (0.84-1.14)
Vegans	88 (27.9)	17 (19.3)	0.67 (0.41-1.09)
			P-het=0.45
**Diabetes**[6]			
Meat eaters	707 (10.0)	446 (63.1)	1.00 (ref)
Fish eaters	75 (14.8)	41 (54.7)	0.78 (0.56-1.08)
Vegetarians	119 (10.6)	84 (70.6)	1.05 (0.81-1.35)
Vegans	7 (13.2)	6 (85.7)	1.07 (0.45-2.51)
			P-het=0.46
**Thyroid disease**[7]			
Meat eaters	1545 (13.2)	1191 (77.1)	1.00 (ref)
Fish eaters	380 (11.6)	273 (71.8)	0.95 (0.83-1.09)
Vegetarians	465 (11.2)	337 (72.5)	0.97 (0.85-1.10)
Vegans	56 (11.8)	37 (66.1)	0.88 (0.63-1.22)
			P-het=0.78

1. Refers to medication use for most the past four weeks specific to the condition described, among participants who reported diagnosis for the condition on the second (10 year) follow-up questionnaire.
2. Adjusted for the cross-classification of sex and age at follow-up (<40, 40-44, 45-49, 50-54, 55-59, 60-64, 65-69, 70-74, ≥75 years), region of residence (eight regions), self-reported current health (excellent, good, fair, poor, unknown), years since reported diagnosis (calculated as year of follow-up questionnaire completion minus reported year of diagnosis; <2, 2-3, 4-5, 6-9, ≥10 years, unknown), and number of self-reported illnesses or conditions (1, 2, 3, ≥4).
3. Reported use of at least one of amlodipine, enalapril, frusemide, propranol, atenolol, bendrofluazide, lisinopril and nifedipine.
4. Reported use of at least one of atorvastatin and simvastatin.
5. Reported use of at least one of beclomethasone and salbutamol.
6. Reported use of at least one of insulin and metformin.
7. Reported use of thyroxine.

For peer review only - http://bmjopen.bmj.com/site/about/guidelines.xhtml

REFERENCES

1 Davey GK, Spencer EA, Appleby PN, *et al.* EPIC-Oxford: lifestyle characteristics and nutrient intakes in a cohort of 33 883 meat-eaters and 31 546 non meat-eaters in the UK. *Public Health Nutr* 2003;**6**:259–68. doi:10.1079/PHN2002430

2 Appleby PN, Davey GK, Key TJ. Hypertension and blood pressure among meat eaters, fish eaters, vegetarians and vegans in EPIC-Oxford. *Public Health Nutr* 2002;**5**:645–54. doi:10.1079/PHN2002332

3 Wang F, Zheng J, Yang B, *et al.* Effects of Vegetarian Diets on Blood Lipids: A Systematic Review and Meta-Analysis of Randomized Controlled Trials. *J Am Heart Assoc* 2015;**4**:e002408. doi:10.1161/JAHA.115.002408

4 Key TJ, Appleby PN, Crowe FL, *et al.* Cancer in British vegetarians: Updated analyses of 4998 incident cancers in a cohort of 32,491 meat eaters, 8612 fish eaters, 18,298 vegetarians, and 2246 vegans. *Am J Clin Nutr* 2014;**100**:378–85. doi:10.3945/ajcn.113.071266

5 Tantamango-Bartley Y, Jaceldo-Siegl K, Fan J, *et al.* Vegetarian Diets and the Incidence of Cancer in a Low-risk Population. *Cancer Epidemiol Biomarkers Prev* 2013;**22**:286–94. doi:10.1158/1055-9965.EPI-12-1060

6 Crowe FL, Appleby PN, Travis RC, *et al.* Risk of hospitalization or death from ischemic heart disease among British vegetarians and nonvegetarians: results from the EPIC-Oxford cohort study. *Am J Clin Nutr* 2013;**97**:597–603. doi:10.3945/ajcn.112.044073

7 Appleby PN, Crowe FL, Bradbury KE, *et al.* Mortality in vegetarians and comparable nonvegetarians in the United Kingdom. *Am J Clin Nutr* 2016;**103**:218–30.

For peer review only

For peer review only - http://bmjopen.bmj.com/site/about/guidelines.xhtml

doi:10.3945/ajcn.115.119461

8 Alewaeters K, Clarys P, Hebbelinck M, *et al.* Cross-sectional analysis of BMI and some lifestyle variables in Flemish vegetarians compared with non-vegetarians. *Ergonomics* 2005;**48**:1433–44. doi:10.1080/00140130500101031

9 Lagerlund M, Drake I, Wirfält E, *et al.* Health-related lifestyle factors and mammography screening attendance in a Swedish cohort study. *Eur J Cancer Prev* 2015;**24**:44–50. doi:10.1097/CEJ.0000000000000025

10 Ibrayev Y, Oda K, Fraser GE, *et al.* Utilization of prostate cancer screening according to dietary patterns and other demographic variables. The adventist health study-2. *J Cancer* 2013;**4**:416–26. doi:10.7150/jca.6442

11 National Health Service. NHS Choices: Breast cancer screening. 2015.http://www.nhs.uk/conditions/breast-cancer-screening/Pages/Introduction.aspx (accessed 27 Feb2017).

12 National Health Service. NHS Choices: Cervical screening. 2015.http://www.nhs.uk/Conditions/Cervical-screening-test/Pages/Introduction.aspx#main-navigation (accessed 27 Feb2017).

13 National Health Service. NHS Choices: Prostate Cancer -PSA testing. 2015.http://www.nhs.uk/Conditions/Cancer-of-the-prostate/Pages/Prevention.aspx (accessed 27 Feb2017).

14 Jepson RG, Hewison J, Thompson A, *et al.* Patient perspectives on information and choice in cancer screening: A qualitative study in the UK. *Soc Sci Med* 2007;**65**:890–9. doi:10.1016/j.socscimed.2007.04.009

For peer review only - http://bmjopen.bmj.com/site/about/guidelines.xhtml

15 Jensen LF, Mukai TO, Andersen B, *et al.* The association between general practitioners' attitudes towards breast cancer screening and women's screening participation. *BMC Cancer* 2012;**12**:254. doi:10.1186/1471-2407-12-254

16 Tatham KC, Patel KP. Suitability of common drugs for patients who avoid animal products. *BMJ* 2014;**348**:g401. doi:10.1136/bmj.g401

17 Beral V, Banks E, Bull D, *et al.* Breast cancer and hormone replacement therapy in the Million Women Study. *Lancet* 2003;**362**:419–27. doi:10.1080/13697130400014698

18 Rossouw JE, Anderson GL, Prentice RL, *et al.* Risks and benefits of estrogen plus progestin in healthy postmenopausal women: principal results From the Women's Health Initiative randomized controlled trial. *JAMA* 2002;**288**:321–33.http://www.ncbi.nlm.nih.gov/pubmed/12117397

For peer review only

For peer review only - http://bmjopen.bmj.com/site/about/guidelines.xhtml

STROBE Statement—checklist of items that should be included in reports of observational studies

	Item No	Recommendation
**Title and abstract**	1	(*a*) Indicate the study's design with a commonly used term in the title or the abstract P.1
		(*b*) Provide in the abstract an informative and balanced summary of what was done and what was found P.2
**Introduction**		
Background/rationale	2	Explain the scientific background and rationale for the investigation being reported P.4
Objectives	3	State specific objectives, including any prespecified hypotheses P.5 Lines 66-68
**Methods**		
Study design	4	Present key elements of study design early in the paper P.5 Lines 72, P.7 Lines 114-116
Setting	5	Describe the setting, locations, and relevant dates, including periods of recruitment, exposure, follow-up, and data collection P.5-7 Lines 71-111
Participants	6	(*a*) *Cohort study*—Give the eligibility criteria, and the sources and methods of selection of participants. Describe methods of follow-up<br>*Case-control study*—Give the eligibility criteria, and the sources and methods of case ascertainment and control selection. Give the rationale for the choice of cases and controls<br>*Cross-sectional study*—Give the eligibility criteria, and the sources and methods of selection of participants P.5 Lines 75-79
		(*b*) *Cohort study*—For matched studies, give matching criteria and number of exposed and unexposed<br>*Case-control study*—For matched studies, give matching criteria and the number of controls per case
Variables	7	Clearly define all outcomes, exposures, predictors, potential confounders, and effect modifiers. Give diagnostic criteria, if applicable P.6-8 Lines 87-111, 129-131, 134-136, 140-142
Data sources/ measurement	8*	For each variable of interest, give sources of data and details of methods of assessment (measurement). Describe comparability of assessment methods if there is more than one group P.5-7 Lines 75-111
Bias	9	Describe any efforts to address potential sources of bias P.7 Lines 119-124
Study size	10	Explain how the study size was arrived at P.5 Lines 76-79, P.7 Lines 117-119
Quantitative variables	11	Explain how quantitative variables were handled in the analyses. If applicable, describe which groupings were chosen and why P.7-8 Lines 129-131, 134-136, 140-142
Statistical methods	12	(*a*) Describe all statistical methods, including those used to control for confounding P.7-8 Lines 114-146
		(*b*) Describe any methods used to examine subgroups and interactions P.8 Lines 143-144
		(*c*) Explain how missing data were addressed P.7-9 Lines 132, 142
		(*d*) *Cohort study*—If applicable, explain how loss to follow-up was addressed<br>*Case-control study*—If applicable, explain how matching of cases and controls was addressed

For peer review only - http://bmjopen.bmj.com/site/about/guidelines.xhtml

Cross-sectional study—If applicable, describe analytical methods taking account of sampling strategy P.7 Lines 114-116

(*e*) Describe any sensitivity analyses P.8 Lines 143-145

Continued on next page

For peer review only

For peer review only - http://bmjopen.bmj.com/site/about/guidelines.xhtml

**Results**		
Participants	13*	(a) Report numbers of individuals at each stage of study—eg numbers potentially eligible, examined for eligibility, confirmed eligible, included in the study, completing follow-up, and analysed P.8-9 Lines 150-153, 159-160
		(b) Give reasons for non-participation at each stage P.8 Lines 150-151
		(c) Consider use of a flow diagram
Descriptive data	14*	(a) Give characteristics of study participants (eg demographic, clinical, social) and information on exposures and potential confounders P.8-9 Lines 150-156
		(b) Indicate number of participants with missing data for each variable of interest P.8-9 Lines 150-153, 159-160
		(c) *Cohort study*—Summarise follow-up time (eg, average and total amount)
Outcome data	15*	*Cohort study*—Report numbers of outcome events or summary measures over time
		Case-control study—Report numbers in each exposure category, or summary measures of exposure
		Cross-sectional study—Report numbers of outcome events or summary measures P.17-19
Main results	16	(*a*) Give unadjusted estimates and, if applicable, confounder-adjusted estimates and their precision (eg, 95% confidence interval). Make clear which confounders were adjusted for and why they were included P.17-19
		(*b*) Report category boundaries when continuous variables were categorized P.17-19
		(*c*) If relevant, consider translating estimates of relative risk into absolute risk for a meaningful time period
Other analyses	17	Report other analyses done—eg analyses of subgroups and interactions, and sensitivity analyses P.10 Lines 179-182
**Discussion**		
Key results	18	Summarise key results with reference to study objectives P.10 Lines 185-194
Limitations	19	Discuss limitations of the study, taking into account sources of potential bias or imprecision. Discuss both direction and magnitude of any potential bias P.14 Lines 275-280
Interpretation	20	Give a cautious overall interpretation of results considering objectives, limitations, multiplicity of analyses, results from similar studies, and other relevant evidence P.10-14
Generalisability	21	Discuss the generalisability (external validity) of the study results P.10-14
**Other information**		
Funding	22	Give the source of funding and the role of the funders for the present study and, if applicable, for the original study on which the present article is based) P.14 Lines 294-296

*Give information separately for cases and controls in case-control studies and, if applicable, for exposed and unexposed groups in cohort and cross-sectional studies.

Note: An Explanation and Elaboration article discusses each checklist item and gives methodological background and published examples of transparent reporting. The STROBE checklist is best used in conjunction with this article (freely available on the Web sites of PLoS Medicine at http://www.plosmedicine.org/, Annals of Internal Medicine at http://www.annals.org/, and Epidemiology at http://www.epidem.com/). Information on the STROBE Initiative is available at www.strobe-statement.org.

For peer review only

BMJ Open

Cross-sectional analyses of participation in cancer screening and use of hormone replacement therapy and medications in meat eaters and vegetarians: the EPIC-Oxford study.

Journal:	*BMJ Open*
Manuscript ID	bmjopen-2017-018245.R1
Article Type:	Research
Date Submitted by the Author:	19-Oct-2017
Complete List of Authors:	Tong, Tammy Yin Ni; Cancer Epidemiology Unit,<br>Appleby, P; Cancer Epidemiology Unit, University of Oxford<br>Bradbury, Kathryn; Cancer Epidemiology Unit, University of Oxford<br>Key, Timothy; Cancer Epidemiology Unit, University of Oxford
**Primary Subject Heading**:	Epidemiology
Secondary Subject Heading:	Nutrition and metabolism, Oncology
Keywords:	NUTRITION & DIETETICS, ONCOLOGY, HEALTH SERVICES ADMINISTRATION & MANAGEMENT

SCHOLARONE™ Manuscripts

For peer review only

Cross-sectional analyses of participation in cancer screening and use of hormone replacement therapy and medications in meat eaters and vegetarians: the EPIC-Oxford study.

Tammy Y.N. Tong[1], Paul N. Appleby[1], Kathryn E. Bradbury[1], Timothy J. Key[1]

1. Cancer Epidemiology Unit, Nuffield Department of Population Health, University of Oxford, UK

Corresponding author: Tammy Y.N. Tong

Postal address: Cancer Epidemiology Unit, University of Oxford, Richard Doll Building, Old Road Campus, Oxford, OX3 7LF

Tel: +44 (0) 1865 289653

Email: tammy.tong@ceu.ox.ac.uk

Word count: 3599 words

Keywords: vegetarians; cancer screening; hormone replacement therapy; medication use

For peer review only - http://bmjopen.bmj.com/site/about/guidelines.xhtml

ABSTRACT

Objectives: To examine differences in health-related behaviours such as screening or testing for cancer, use of hormone replacement therapy (HRT), and use of other medications in different diet groups.

Design: We studied 31,260 participants across four diet groups (18,155 meat eaters, 5,012 fish eaters, 7,179 vegetarians, 914 vegans) in the UK EPIC-Oxford cohort. Information was collected in 5 (around 2000-2003) or 10 (around 2007) year follow-up questionnaires regarding participation in breast screening, cervical screening, prostate specific antigen (PSA) testing, use of HRT, and use of medications for the past four weeks. Using Poisson regression, we estimated the prevalence ratios (PR) for each behaviour across people of different diet groups, using meat-eaters as the reference group.

Results: Compared with meat-eaters, vegetarian (PR; 95% confidence interval: 0.94; 0.89, 0.98) and vegan (0.82; 0.71, 0.95) women reported lower participation in breast screening, and vegetarian men were less likely to report PSA testing (0.82; 0.71, 0.96). No differences were observed among women for cervical screening. In women, all non-meat eating groups reported lower use of HRT compared with meat-eaters (p heterogeneity<0.0001). Lower reported use of any medication was observed for participants in all non-meat eating groups with no (p<0.0001) or one (p=0.0002) self-reported illness. No heterogeneity was observed across the diet groups for the reported use of specific medication for high blood pressure, high blood cholesterol, asthma, diabetes, and thyroid disease.

Conclusions: Differences in self-reported breast screening, PSA testing, HRT use and overall medication use were observed across the diet groups. Whether such differences contribute to differential long-term disease risks requires further study.

For peer review only - http://bmjopen.bmj.com/site/about/guidelines.xhtml

Strengths and limitations of this study

- This study is the first to simultaneously examine the reported uptake of breast and cervical cancer screening, prostate specific antigen testing, hormone replacement therapy (HRT) use and medication use in different diet groups.
- The study includes a large number of participants recruited from across different regions in the United Kingdom, with a high proportion of fish eaters, vegetarians, and vegans.
- Recall bias is possible because assessment of cancer screening or testing, HRT use and medication use was based on self-report, although there is no indication that such misclassification bias should differ by diet group.
- The study is cross-sectional and we cannot infer causality.

For peer review only

For peer review only - http://bmjopen.bmj.com/site/about/guidelines.xhtml

INTRODUCTION

People of different habitual diet groups have been shown to have different health characteristics. Compared to meat eaters, vegetarians generally have lower BMI, blood pressure, and circulating low density lipoprotein cholesterol levels [1–3], characteristics likely to reduce disease risk. However, evidence on the long-term risk of many non-communicable diseases across people of different diet groups is limited.

For cancer risk, both a United Kingdom (UK) [4] and a United States (US) [5] study reported lower risk of overall cancer incidence with a vegetarian diet. Because health related behaviours, such as participation in cancer screening [6] or use of hormone replacement therapy (HRT) [7,8], may contribute to the observed rates of cancer, the presence of any differences in these behaviours between diet groups in different populations deserve further investigation. Results from a Swedish cohort [9] and a US cohort [10] showed that vegetarians (including vegans and people who ate fish but not meat) had lower odds of attending breast screening and prostate cancer screening respectively, when compared with meat eaters, and vegetarians also had lower use of HRT compared with non-vegetarians [5].

For cardiovascular diseases, vegetarians in EPIC-Oxford have been observed to have lower ischaemic heart disease risk (hospitalization and death combined) [11], but no significant difference in ischaemic heart disease mortality was observed between diet groups in the same population [12]. The reason for this apparent difference between incidence and mortality is unclear. One possible explanation could be the differential use of appropriate medications in the different diet groups, which subsequently influences disease mortality. In a Belgian population for example, vegetarians had lower use of prescription medications compared to non-vegetarians, but similar use of non-prescription drugs [13].

For peer review only - http://bmjopen.bmj.com/site/about/guidelines.xhtml

The increasing popularity and interest in vegetarian diets [14] prompts research on the long-term health of vegetarians and vegans. Because health behaviour such as screening or medication use may ultimately influence disease risk, the understanding of any differences in these behaviours by diet group is crucial for the appropriate appraisal of possible differences in disease risk between diet groups. However, current knowledge on this topic is insufficient, because literature on participation in screening and use of medication across people of different diet groups is scarce. Therefore, the aim of this study was to assess some of these relevant health behaviours, including participation in cancer screening or testing, and use of HRT and other medications among people of different diet groups, in a large population-based cohort in the UK with a high percentage of vegetarians.

METHODS

Study population

The EPIC-Oxford study is a UK based cohort recruited between 1993 and 1999. The study protocol was approved by a Multicentre Research Ethics Committee (Scotland A Research Ethics Committee) and participants gave written informed consent. Details of the recruitment process have been described previously [1]. In brief, a combination of general practitioner (GP) recruitment and postal recruitment was used. The GP recruitment invited men and women aged 35 to 59 years registered with participating GPs and recruited 7,421 participants. The postal recruitment was targeted at vegetarians, vegans, and other people interested in diet and health, by contacting members of The Vegetarian Society, The Vegan Society, and via leaflets enclosed in vegetarian and health food magazines and displayed in health-food shops, and recruited 57,990 participants aged ≥20 years. Altogether, 57,443 participants completed a full recruitment questionnaire which asked about their personal details (including postcode to

For peer review only - http://bmjopen.bmj.com/site/about/guidelines.xhtml

For peer review only

which a Townsend index of area-level deprivation was assigned [15]), habitual diet and other health and lifestyle characteristics, including personal and family medical history, medication use, socio-economic characteristics, smoking and drinking behaviour, and physical activity levels. A follow-up questionnaire was sent to surviving participants approximately 5 years after recruitment (mostly from 2000 to 2003), and a second follow-up questionnaire was mailed approximately 10 years after recruitment (mostly in 2007). In the follow-up questionnaires, updated information was gathered on diet, health and lifestyle, including self-reported current health.

Assessment of diet group

In the recruitment questionnaire and each subsequent follow-up questionnaire, four questions were asked regarding consumption of meat, fish, dairy products, and eggs, in the form of "Do you eat any meat?" or similar for the other three food groups. Responses to these questions were used to assign participants to one of four diet groups at each time point: meat eaters (participants who ate meat, irrespective of whether they ate fish, dairy products or eggs); fish eaters (participants who did not eat meat but did eat fish); vegetarians (participants who did not eat meat or fish, but did eat one or both of dairy products and eggs), and vegans (participants who did not eat meat, fish, dairy products, or eggs).

Assessment of participation in screening, HRT and medication use

In the follow-up questionnaires, women were asked if they had ever had a breast screening by mammography, cervical screening by the smear test (only on the 5 year follow-up questionnaire), or used HRT, and men were asked if they had ever had a prostate specific

For peer review only - http://bmjopen.bmj.com/site/about/guidelines.xhtml

antigen (PSA) test (only on the 10 year follow-up questionnaire). On the 10 year follow-up questionnaire, all participants were asked if they had used any medication for most of the last four weeks, with 36 named medications and a free text field for reporting regular use of any medication not on the list; participants were also asked if they had been diagnosed with any of a list of 29 medical conditions, and the year when the condition was first diagnosed. The full list of the 36 medications and 29 medical conditions is given in Supplementary text 1 and 2. The corresponding question on medication use on the 5 year questionnaire was shorter, with 20 named medications and 26 medical conditions.

For assessment of specific medication use, five common medical conditions associated with specific medications were identified: high blood pressure (commonly treated with one or more of amlodipine, enalapril, frusemide, propranolol, atenolol, bendrofluazide, lisinopril and nifedipine), high blood cholesterol (atorvastatin and simvastatin), asthma (beclomethasone and salbutamol), diabetes (insulin and metformin), and thyroid disease (thyroxine).

Statistical analyses

Information on assignment to diet group and assessment of health behaviour from the 10 year follow-up questionnaire was used for our analyses, except for the assessment of participation in cervical screening which was only asked on the 5 year follow-up questionnaire. Participants were excluded from all analysis if they did not answer the relevant questions to be assigned to an appropriate diet group (n=28), and in order to ensure that an overlapping population was used for the analyses of all outcomes, they were also excluded if they did not answer the relevant question on medication use (n=407). For the analyses related to participation in breast screening, cervical screening, PSA testing or HRT use, only women or men who answered the relevant question and were in the specified age group at questionnaire

For peer review only - http://bmjopen.bmj.com/site/about/guidelines.xhtml

completion were included. The age group specifications were as follows: age 50 to 74 years for breast screening, age 25 to 74 years for cervical screening, age 50 to 84 years for PSA testing, and age 50 to 74 years for HRT use. For HRT use, we further restricted the analysis to post-menopausal women, determined by including only participants who answered yes to the question 'Have you been through your menopause?' on the follow-up questionnaire.

For each analysis, we used Poisson regression to estimate prevalence ratios (95% confidence intervals, CI) of cancer screening or testing (breast screening, cervical screening, PSA testing), HRT use, or medication use in different diet groups, using meat eaters as the reference group. For analyses of cancer screening or testing and use of HRT, we adjusted for age at follow-up (<40, 40-44, 45-49, 50-54, 55-59, 60-64, 65-69, 70-74, ≥75 years as appropriate for the age range included in the analysis), region of recruitment (eight geographical regions across the UK), and self-reported current health (excellent, good, fair, poor, unknown). For analyses of any medication use, we adjusted for the cross-stratification of sex and age at follow-up, region of recruitment, self-reported current health, and the number of self-reported illnesses or conditions (0, 1, 2, 3, ≥4). To further assess whether any variation in medication use by diet group varied by health status, we repeated the analyses stratified by the number of self-reported illnesses or conditions using the above categorisation. Subsequently, for each of high blood pressure, high blood cholesterol, asthma, diabetes, and thyroid disease, we estimated the prevalence ratios of taking appropriate medication by diet group among people diagnosed with each condition in turn, adjusting for covariates as above and additionally for years since reported diagnosis, calculated as year of follow-up questionnaire completion minus reported year of diagnosis (<2, 2-3, 4-5, 6-9, ≥10 years, unknown).

As sensitivity analyses, we repeated the analyses as follows: using data from the 5 year follow-up questionnaire where available; and further adjusting for smoking status (never,

For peer review only - http://bmjopen.bmj.com/site/about/guidelines.xhtml

former, current, unknown), alcohol consumption (<1 g/day, 1-7 g/day, 8-15 g/day, ≥16 g/day), Townsend index of area-level deprivation (quartiles and unknown), and education level (no qualifications, basic secondary e.g. O level, higher secondary e.g. A level, degree, unknown). All statistical analyses were performed using Stata release 14.1 (StataCorp), and *P* values < 0.05 were considered statistically significant.

RESULTS

Cohort characteristics

Overall, 57,443 participants in EPIC-Oxford cohort completed a full recruitment questionnaire, of whom 38,043 (66%) completed the 5 year follow-up questionnaire, and 31,695 (55%) completed the 10 year follow-up questionnaire. After excluding participants who did not answer the relevant questions on diet group or on medication use, data for 31,260 participants who completed the 10 year follow-up questionnaire (18,155 meat eaters, 5,012 fish eaters, 7,179 vegetarians, and 914 vegans) were used for most of the analyses. Characteristics of the participants are presented in **Table 1**. Overall, non-meat eaters were younger, more likely to report having excellent health, less likely to report taking medication in the past four weeks, and less likely to have reported any illnesses or conditions.

Participation in screening and use of HRT and medications

Overall, 14,016 women were included in the analyses for breast screening, 27,781 women for cervical screening, and 4,783 men for PSA testing (**Table 2**). In women, compared with meat eaters, vegetarians (prevalence ratio; 95% CI: 0.94; 0.89, 0.98) and vegans (0.82; 0.71, 0.95), but not fish eaters (0.96; 0.92, 1.01) had lower reported attendance of breast screening, but no

For peer review only - http://bmjopen.bmj.com/site/about/guidelines.xhtml

significant heterogeneity was observed between the diet groups for reported participation in cervical screening (*P*-heterogeneity=0.37). In men, vegetarians had lower reported uptake of PSA testing (0.82; 0.71, 0.96) than meat eaters, while the difference in uptake appeared lower but did not reach statistical significance in vegans (0.72; 0.50, 1.02), and was not significantly different in fish eaters (0.99; 0.85, 1.07). For HRT use, women who were non-meat eaters reported lower use (fish eaters: 0.80; 0.73, 0.88; vegetarians: 0.74; 0.68, 0.81; vegans: 0.42; 0.30, 0.60) compared with women who were meat eaters (**Table 3**).

Irrespective of the number of self-reported illnesses and conditions, non-meat eaters reported lower use of any medication (fish eaters: 0.92; 0.87, 0.96; vegetarians: 0.93; 0.89, 0.98; vegans 0.71; 0.63, 0.81) compared with meat eaters (**Table 4**). When the analyses were stratified by the number of self-reported illnesses or conditions, non-meat eaters with no ($P<0.0001$) or one ($P=0.0002$) illness or condition reported lower medication use compared with meat eaters, but the association was attenuated and no longer statistically significant among participants with two, three, or four or more illnesses or conditions. For medication use specific to several common illnesses and conditions, no significant differences were observed between the diet groups in the reported use of appropriate medications for high blood pressure, high blood cholesterol, asthma, diabetes, or thyroid disease, among participants diagnosed with each of these conditions (**Table 5**). Results were consistent when we repeated the analyses where possible using data from the 5 year follow-up questionnaire, or when we further adjusted for smoking, alcohol consumption, Townsend deprivation index, and education level (results not shown).

For peer review only - http://bmjopen.bmj.com/site/about/guidelines.xhtml

DISCUSSION

Summary of results

In this UK population-based cohort with a large proportion of participants from different diet groups, we generally observed lower participation in breast screening and lower HRT use among women who were non-meat eaters (separately categorised as fish eaters, vegetarians, and vegans) compared with women who were meat eaters. Vegetarian men had lower participation in PSA testing compared with meat eating men, but no significant difference was observed for cervical screening in women across the diet groups. For medication use, non-meat eaters were less likely to report taking medications than meat eaters overall, but there were no significant differences in medication use among people reporting two or more illnesses or conditions, or for people reporting taking specific medications for various self-reported conditions.

Comparison with other studies

Few studies have reported on the participation in cancer screening or testing, HRT use or medication use among people of different diet groups, and no study has assessed all these behaviours simultaneously in the same cohort. For breast cancer screening, consistent with our findings, the Swedish Malmö Diet and Cancer Study reported that non-attendance for breast cancer screening was more likely in people who were vegetarians or vegans (odds ratio or OR; 95% confidence interval: 1.49; 1.11, 1.99) [9]. Analyses of data from the Adventist Health Study-2 in the United States and Canada showed that all non-meat eaters were less likely to report PSA testing compared with meat eaters (0.79; 0.66, 0.95 for fish eaters; 0.76; 0.67, 0.86 for vegetarians; and OR 0.50; 0.42, 0.60 for vegans) [10], whereas we only

For peer review only - http://bmjopen.bmj.com/site/about/guidelines.xhtml

observed a lower reported uptake among the vegetarians but not the fish eaters (nor the vegans, perhaps because of limited numbers) compared with meat eaters in EPIC-Oxford. However, given the much higher rates of PSA testing in the Adventist Health Study-2 (73.3% versus 31.5% in EPIC-Oxford), attitudes towards screening are likely to be different in the two populations, and therefore the results might not be directly comparable. Similar to our study, the Adventist Health Study-2 also reported lower ever use of HRT (adjusted for age and race) in pesco-vegetarians (21.0%) and lacto-vegetarians (20.4%), and the lowest use in vegans (16.2%), when compared with non-vegetarians (22.4%) [5].

For medication use, a cross-sectional study in a Belgian population reported lower use of prescribed medications when comparing vegetarians to a reference Belgian population (25.5% versus 47.3%, p<0.001) [13]. While this is consistent with our findings on overall medication use, the study did not assess the use of medications stratified by the number of illnesses, nor did they assess appropriate medication use for specific medical conditions. No studies were found which examined participation of cervical screening among people of different diet groups.

For peer review only

Interpretation of findings and implications

Our findings indicate differences in some health related behaviours between people of different diet groups, although the reasons behind such differences are unclear. For the observed differences in screening rates, possible explanations could be related to different attitudes towards the screening programmes. In the UK since 1988 [6,16], all women aged 50 to 70 are invited to attend breast cancer screening clinics [17] and all women aged 25 to 64 are invited for cervical screening [18] at regular intervals. On the other hand, there is no national programme for PSA testing, although men over the age of 50 are eligible to arrange

For peer review only - http://bmjopen.bmj.com/site/about/guidelines.xhtml

for testing via their GP if they wish [19]. In studies which assessed attitudes towards cancer screening or testing, common reasons which affect people's participation in screening include their education level and knowledge of the procedure, recommendation by their doctor, fear of the procedure or the outcome, or their perceived risk of cancer [20–23]. If vegetarians and vegans felt their diets or lifestyles were protective against cancer for example, they might be more likely to forgo cancer screening as a result of lower perceived risk. However, no information was found on whether or how such attitudes may vary by diet group.

In a small focus group study in Scotland which asked participants about their attitudes towards cancer screening (n=31 for cervical screening, n=10 for breast screening), the study participants reported that they felt pressure from health care professionals, family and friends to attend cervical screening but not breast screening, and that they also considered cervical screening to be normative routine behaviour [24]. Such differences in attitudes towards breast screening and cervical screening are of interest, as this may help to explain the differences we observed in participation for breast screening but not cervical screening, if the latter was considered routine behaviour. However, relevant evidence is lacking, and both dietary and non-dietary factors which are associated with attendance for either breast screening or PSA testing deserve further study.

Reasons for the observed lower prevalence of HRT use and medication use among people of different diet groups are also unclear. The prevalence of medication use in meat eaters (56%) in EPIC-Oxford was slightly higher than the UK average of 43% of men and 50% of women aged 16 or above who reported taking at least one prescribed medicine in the last week [25], confirming the relatively low prevalence of medication use in the vegetarians (39%) and vegans (28%). However, given the differences in age ranges and possible differences in medications accounted for, strict comparisons cannot be made. Because lower reported use of medications was observed even in people with no (especially) or only one reported illness or

For peer review only - http://bmjopen.bmj.com/site/about/guidelines.xhtml

condition, better health among non-meat eaters is unlikely to be the only, or a sufficient explanation for the differences. Non-meat eaters may also be reluctant to take medications which are likely to contain animal-derived products [26], or may prefer to use homoeopathic medications [13] or other alternative therapies. Since information on medication use in this study was based on a pre-specified list from the follow-up questionnaire, it was not possible to assess the use of alternative therapies or any other named medications, despite their possible contributions to prevalence of overall medication use.

Differential participation in screening for breast or prostate cancer, use of HRT, and use of medications for people of distinct diet groups may ultimately lead to differences in disease incidence or prognosis due to possible detection bias and differential post diagnosis treatment. For example, breast cancer screening results in higher incidence but reduced mortality from breast cancer among those who are screened [6]. Prostate cancer testing is also linked to increased incidence in those who are tested [27,28]. Therefore, using breast cancer as an example, given the lower rates of breast cancer screening among non-meat eating women both in EPIC-Oxford and in the Swedish Malmö Diet and Cancer Study [9], it is possible that the observed incidence of breast cancer in these diet groups underestimates the true incidence owing to detection bias, but that ultimately these women would be expected to have a somewhat higher mortality from breast cancer. Therefore, future work on assessing breast cancer risk in people of different diet groups should take into account any differences in screening rates between diet groups.

Similarly, it is not clear why there was differential use of HRT in the four diet groups, for example whether it was because non-meat eaters were less likely to have symptoms, or because they were less likely to seek treatment when symptoms appear. Regardless of the underlying reason, the observed lower reported use of HRT among non-meat eating women deserves attention, because use of HRT may confound any observed associations between

For peer review only - http://bmjopen.bmj.com/site/about/guidelines.xhtml

diet group and breast cancer, given that HRT preparations containing oestrogens and progestogens have been shown to increase the risk of breast cancer [7,8].

Overall, our findings showed some differences in health related behaviours between people of different diet groups, thereby highlighting the need to consider such differences when conducting longitudinal analyses in these populations. Further study is warranted to understand why people of different diet groups have differential participation in breast screening or prostate cancer testing, HRT use, and overall medication use, and whether or how these differences are related to future disease risk.

Strengths and limitations

This study is the first to simultaneously examine participation in cancer screening or testing, HRT use and medication use in different diet groups. A strength of the study is the large sample size recruited from across different regions in the UK. Additionally, information was collected on a range of factors which may also be associated with the behaviours of interest, allowing adjustment for these factors. Of potential limitations, recall bias is possible because assessment of the behaviours of interest (i.e. breast screening, PSA testing, HRT use and overall medication use) as well as existing medical conditions was based on self-report, although there is no indication that such misclassification bias should differ by diet group. The reasons for which people adhered to each diet group were not recorded, although such reasons may be relevant to the other health behaviours studied. Because of the relatively small number of vegans in our study sample, the role of chance in explaining the findings relating to this diet group, especially subgroup analyses related to medication use, cannot be ruled out. As with most population cohorts, some degree of self-selection and healthy cohort bias may also be present.

For peer review only - http://bmjopen.bmj.com/site/about/guidelines.xhtml

CONCLUSIONS

In this population, we observed differences in breast screening, PSA testing, HRT use and overall medication use between meat eaters, fish eaters, vegetarians and vegans, but no significant differences between diet groups for cervical screening, or medication use in people with two or more illnesses or for specific conditions. The reasons for these differences require further investigation. Nonetheless, such differences may be related to or could confound any differences in observed morbidity or mortality from cancer and other diseases between people of different diet groups, and therefore should be considered in future epidemiological studies.

STATEMENTS

Acknowledgements

We thank all participants in the EPIC-Oxford cohort for their invaluable contribution. The work is supported by the UK Medical Research Council MR/M012190/1 and Cancer Research UK 570/A16491 and C9221/A19170. KEB is supported by the Girdlers' New Zealand Health Research Council Fellowship. TJK is a member of the Vegan Society; the other authors had no conflicts of interest.

Availability of data and materials

The data access policy for EPIC-Oxford is available via the study website (http://www.epic-oxford.org/data-access-sharing-and-collaboration/).

For peer review only - http://bmjopen.bmj.com/site/about/guidelines.xhtml

Author's contributions

TYNT, PNA, and TJK conceived and designed the research question. TYNT and PNA analysed the data. TYNT wrote the first draft of the manuscript, and PNA, KEB and TJK provided input on data analysis and interpretation of results. All authors revised the manuscript critically for important intellectual content, and read and approved the final manuscript.

For peer review only

For peer review only - http://bmjopen.bmj.com/site/about/guidelines.xhtml

Table 1 Characteristics by diet group of participants in the EPIC-Oxford study who completed the second follow-up questionnaire (n=31260) [1].

Characteristic	Meat eaters	Fish eaters	Vegetarians	Vegans	Total
Number of participants (% female)	18155 (78.2)	5012 (81.8)	7179 (76.3)	914 (66.1)	31260 (78.0)
Mean (SD) age at questionnaire completion, years	58.9 (12.5)	53.8 (12.5)	51.6 (12.7)	50.7 (12.3)	56.1 (13.0)
Smoking status[2], n (%)					
Never smoker	10073 (55.7)	2786 (55.6)	4339 (60.5)	547 (59.9)	17745 (56.9)
Former smoker	6927 (38.3)	1961 (39.2)	2460 (34.3)	330 (36.1)	11678 (37.5)
Current smoker	1094 (6.0)	260 (5.2)	367 (5.1)	36 (3.9)	1757 (5.6)
Mean (SD) alcohol consumption, g/d	8.7 (9.3)	8.2 (8.7)	7.6 (8.9)	6.7 (9.2)	8.3 (9.1)
Self-reported current health[2], n (%)					
Excellent	3713 (21.9)	1323 (28.1)	1950 (28.7)	325 (37.2)	7311 (24.9)
Good	9962 (58.8)	2688 (57.0)	3851 (56.6)	446 (51.0)	16947 (57.8)
Fair	2858 (16.9)	612 (13.0)	876 (12.9)	80 (9.2)	4426 (15.1)
Poor	400 (2.4)	92 (2.0)	122 (1.8)	23 (2.6)	637 (2.2)
Townsend deprivation index[2], n (%)					
Richest category	4463 (27.6)	984 (21.8)	1542 (23.7)	153 (18.3)	7141 (25.5)
Poorest category	3438 (21.2)	1207 (26.8)	1732 (26.7)	285 (34.1)	6662 (23.8)
In same diet group at recruitment, n (%)	15908 (87.7)	3057 (61.1)	6373 (89.1)	573 (62.7)	25911 (83.0)
Taking medication in the past 4 weeks, n (%)	10196 (56.2)	2105 (42.0)	2829 (39.4)	255 (27.9)	15385 (49.2)
Number of reported illnesses and conditions, n (%)					
None	4455 (24.5)	1635 (32.6)	2603 (36.3)	344 (37.6)	9037 (28.9)
One	4724 (26.0)	1472 (29.4)	2170 (30.2)	291 (31.8)	8657 (27.7)
Two	3682 (20.3)	906 (18.1)	1261 (17.6)	154 (16.8)	6003 (19.2)
Three	2404 (13.2)	524 (10.5)	630 (8.8)	74 (8.1)	3632 (11.6)
Four or more	2890 (15.9)	475 (9.5)	515 (7.2)	51 (5.6)	3931 (12.6)
Reported high blood pressure[2], n (%)	4397 (29.2)	686 (16.2)	944 (15.2)	85 (10.6)	6112 (23.2)
and taking appropriate medication, n (%)	2573 (58.5)	357 (52.0)	430 (45.6)	40 (47.1)	3400 (55.6)
Reported high blood cholesterol[2], n (%)	3351 (23.1)	561 (13.5)	645 (10.5)	44 (5.5)	4601 (18.0)
and taking appropriate medication, n (%)	1646 (49.1)	209 (37.3)	243 (37.7)	14 (31.8)	2112 (45.9)
Reported asthma[2], n (%)	1885 (13.6)	496 (12.1)	758 (12.4)	88 (11.1)	3227 (12.9)
and taking appropriate medication, n (%)	737 (39.1)	169 (34.1)	246 (32.5)	17 (19.3)	1169 (36.2)
Reported diabetes[2], n (%)	707 (5.2)	75 (1.9)	119 (2.0)	7 (0.9)	908 (3.7)
and taking appropriate medication, n (%)	446 (63.1)	41 (54.7)	84 (70.6)	6 (85.7)	577 (63.5)
Reported thyroid disease[2], n (%)	1545 (11.1)	380 (9.2)	465 (7.6)	56 (7.1)	2446 (9.8)
and taking appropriate medication, n (%)	1191 (77.1)	273 (71.8)	337 (72.5)	37 (66.1)	1838 (75.1)

1. Based on participant characteristics at the time of the second follow-up questionnaire (completed approximately10 years from baseline, around 2007).
2. Unknown for some participants.

For peer review only

For peer review only - http://bmjopen.bmj.com/site/about/guidelines.xhtml

Table 2 Participation in screening by diet group of women and men in the EPIC-Oxford study.

Screening/Diet group	Number answering the relevant question [1]	Number (%) answering in the affirmative [1]	Prevalence ratio (95% CI) [1]
**Breast screening[2]**			
Meat eaters	9239	8813 (95.4)	1.00 (ref)
Fish eaters	2143	1928 (90.0)	0.96 (0.92,1.01)
Vegetarians	2395	2078 (86.8)	0.94 (0.89,0.98)
Vegans	239	182 (76.2)	0.82 (0.71,0.95)
			P-het=0.004
**Cervical screening[3]**			
Meat eaters	15936	15365 (96.4)	1.00 (ref)
Fish eaters	4513	4369 (96.8)	1.00 (0.97,1.03)
Vegetarians	6574	6268 (95.3)	0.98 (0.95,1.01)
Vegans	758	691 (91.2)	0.94 (0.87,1.02)
			P-het=0.37
**Prostate specific antigen testing[4]**			
Meat eaters	3078	1066 (34.6)	1.00 (ref)
Fish eaters	594	181 (30.5)	0.99 (0.85,1.17)
Vegetarians	947	228 (24.1)	0.82 (0.71,0.96)
Vegans	164	33 (20.1)	0.72 (0.50,1.02)
			P-het=0.023

1. Number answering the relevant question and number (%) answering in the affirmative were as observed. Prevalence ratios were adjusted for age at follow-up (<40, 40-44, 45-49, 50-54, 55-59, 60-64, 65-69, 70-74, ≥75 years, as appropriate according to the age range of included participants), region of residence (eight regions), and self-reported current health (excellent, good, fair, poor, unknown).
2. Included women aged 50 to 74 who answered the relevant question on the second (10 year) follow-up questionnaire.
3. Included women aged 25 to 74 who answered the relevant question on the first (5 year) follow-up questionnaire
4. Included men aged 50 to 84 who answered the relevant question on the second (10 year) follow-up questionnaire.

Table 3 Use of hormone replacement therapy by diet group of women in the EPIC-Oxford study.

Diet group	Number answering the relevant question [1]	Number (%) answering in the affirmative [1]	Prevalence ratio (95% CI) [1]
Meat eaters	6911	3098 (44.8)	1.00 (ref)
Fish eaters	1614	541 (33.5)	0.80 (0.73,0.88)
Vegetarians	1778	541 (30.4)	0.74 (0.68,0.81)
Vegans	188	31 (16.5)	0.42 (0.30,0.60)
			P-het<0.0001

1. Number answering the relevant question and number (%) answering in the affirmative were as observed. Prevalence ratios were adjusted for age at follow-up 50-54, 55-59, 60-64, 65-69, 70-74 years), region of residence (eight regions), and self-reported current health (excellent, good, fair, poor, unknown). Included post-menopausal women aged 50 to 74 who answered the relevant question on the second (10 year) follow-up questionnaire.

For peer review only

Table 4 Medication use by number of self-reported illnesses or conditions and diet group of participants in the EPIC-Oxford study. [1]

Number of self-reported illnesses or conditions / Diet group	Number of participants [2]	Percentage taking any medication [2]	Prevalence ratio (95% CI) [2]
**Any number**[3]			
Meat eaters	18155	56.2	1.00 (ref)
Fish eaters	5012	42.0	0.92 (0.87-0.96)
Vegetarians	7179	39.4	0.93 (0.89-0.98)
Vegans	914	27.9	0.71 (0.63-0.81)
			P-het<0.0001
**None**			
Meat eaters	4455	16.9	1.00 (ref)
Fish eaters	1635	11.9	0.80 (0.68-0.94)
Vegetarians	2603	11.5	0.80 (0.70-0.92)
Vegans	344	6.1	0.47 (0.30-0.72)
			P-het<0.0001
**One**			
Meat eaters	4724	48.9	1.00 (ref)
Fish eaters	1472	39.1	0.87 (0.80-0.96)
Vegetarians	2170	40.5	0.91 (0.84-0.99)
Vegans	291	29.2	0.69 (0.55-0.85)
			P-het=0.0002
**Two**			
Meat eaters	3682	66.9	1.00 (ref)
Fish eaters	906	58.8	0.94 (0.86-1.04)
Vegetarians	1261	58.1	0.97 (0.89-1.06)
Vegans	154	42.2	0.74 (0.58-0.95)
			P-het=0.082
**Three**			
Meat eaters	2404	82.6	1.00 (ref)
Fish eaters	524	74.0	0.94 (0.84-1.05)
Vegetarians	630	73.0	0.94 (0.84-1.04)
Vegans	74	59.5	0.78 (0.57-1.05)
			P-het=0.22
**Four or more**			
Meat eaters	2890	93.0	1.00 (ref)
Fish eaters	475	86.9	0.96 (0.86-1.06)
Vegetarians	515	88.9	0.98 (0.89-1.09)
Vegans	51	78.4	0.87 (0.63-1.19)
			P-het=0.70

1. Refers to medication use for most of the past four weeks on the second (10 year) follow-up questionnaire, excluding HRT and contraceptive pills.
2. Number of participants and percentage taking any medication were as observed. Prevalence ratios were adjusted for the cross-classification of sex and age at follow-up (<40, 40-44, 45-49, 50-54, 55-59, 60-64, 65-69, 70-74, ≥75 years), region of residence (eight regions), and self-reported current health (excellent, good, fair, poor, unknown).
3. Prevalence ratios for this category were further adjusted for the number of self-reported illnesses or conditions (0, 1, 2, 3, ≥4).

For peer review only

Table 5 Medication use for specific conditions by diet group of participants in the EPIC-Oxford study.[1]

Condition/ Diet group	Number reporting the condition (mean years since reported diagnosis) [2]	Number (%) taking appropriate medication [2]	Prevalence ratio (95% CI) [2]
**High blood pressure**[3]			
Meat eaters	4397 (9.8)	2573 (58.5)	1.00 (ref)
Fish eaters	686 (9.3)	357 (52.0)	0.97 (0.86-1.08)
Vegetarians	944 (9.0)	430 (45.6)	0.91 (0.82-1.01)
Vegans	85 (9.0)	40 (47.1)	0.92 (0.67-1.26)
			P-het=0.37
**High blood cholesterol**[4]			
Meat eaters	3351 (6.3)	1646 (49.1)	1.00 (ref)
Fish eaters	561 (5.3)	209 (37.3)	0.88 (0.76-1.01)
Vegetarians	645 (5.5)	243 (37.7)	0.94 (0.81-1.08)
Vegans	44 (7.1)	14 (31.8)	0.74 (0.44-1.26)
			P-het=0.20
**Asthma**[5]			
Meat eaters	1885 (25.3)	737 (39.1)	1.00 (ref)
Fish eaters	496 (23.2)	169 (34.1)	0.98 (0.82-1.17)
Vegetarians	758 (23.4)	246 (32.5)	0.97 (0.84-1.14)
Vegans	88 (27.9)	17 (19.3)	0.67 (0.41-1.09)
			P-het=0.45
**Diabetes**[6]			
Meat eaters	707 (10.0)	446 (63.1)	1.00 (ref)
Fish eaters	75 (14.8)	41 (54.7)	0.78 (0.56-1.08)
Vegetarians	119 (10.6)	84 (70.6)	1.05 (0.81-1.35)
Vegans	7 (13.2)	6 (85.7)	1.07 (0.45-2.51)
			P-het=0.46
**Thyroid disease**[7]			
Meat eaters	1545 (13.2)	1191 (77.1)	1.00 (ref)
Fish eaters	380 (11.6)	273 (71.8)	0.95 (0.83-1.09)
Vegetarians	465 (11.2)	337 (72.5)	0.97 (0.85-1.10)
Vegans	56 (11.8)	37 (66.1)	0.88 (0.63-1.22)
			P-het=0.78

1. Refers to medication use for most the past four weeks specific to the condition described, among participants who reported diagnosis for the condition on the second (10 year) follow-up questionnaire.
2. Number reporting the condition (mean years since reported diagnosis) and number (%) taking appropriate medication were as observed. Prevalence ratios were adjusted for the cross-classification of sex and age at follow-up (<40, 40-44, 45-49, 50-54, 55-59, 60-64, 65-69, 70-74, ≥75 years), region of residence (eight regions), self-reported current health (excellent, good, fair, poor, unknown), years since reported diagnosis (calculated as year of follow-up questionnaire completion minus reported year of diagnosis; <2, 2-3, 4-5, 6-9, ≥10 years, unknown), and number of self-reported illnesses or conditions (1, 2, 3, ≥4).
3. Reported use of at least one of amlodipine, enalapril, frusemide, propranolol, atenolol, bendrofluazide, lisinopril and nifedipine.
4. Reported use of at least one of atorvastatin and simvastatin.
5. Reported use of at least one of beclomethasone and salbutamol.
6. Reported use of at least one of insulin and metformin.
7. Reported use of thyroxine.

For peer review only - http://bmjopen.bmj.com/site/about/guidelines.xhtml

REFERENCES

1 Davey GK, Spencer EA, Appleby PN, *et al.* EPIC-Oxford: lifestyle characteristics and nutrient intakes in a cohort of 33 883 meat-eaters and 31 546 non meat-eaters in the UK. *Public Health Nutr* 2003;**6**:259–68. doi:10.1079/PHN2002430

2 Appleby PN, Davey GK, Key TJ. Hypertension and blood pressure among meat eaters, fish eaters, vegetarians and vegans in EPIC-Oxford. *Public Health Nutr* 2002;**5**:645–54. doi:10.1079/PHN2002332

3 Wang F, Zheng J, Yang B, *et al.* Effects of Vegetarian Diets on Blood Lipids: A Systematic Review and Meta-Analysis of Randomized Controlled Trials. *J Am Heart Assoc* 2015;**4**:e002408. doi:10.1161/JAHA.115.002408

4 Key TJ, Appleby PN, Crowe FL, *et al.* Cancer in British vegetarians: Updated analyses of 4998 incident cancers in a cohort of 32,491 meat eaters, 8612 fish eaters, 18,298 vegetarians, and 2246 vegans. *Am J Clin Nutr* 2014;**100**:378–85. doi:10.3945/ajcn.113.071266

5 Tantamango-Bartley Y, Jaceldo-Siegl K, Fan J, *et al.* Vegetarian Diets and the Incidence of Cancer in a Low-risk Population. *Cancer Epidemiol Biomarkers Prev* 2013;**22**:286–94. doi:10.1158/1055-9965.EPI-12-1060

6 Independent UK Panel on Breast Cancer Screening. The benefits and harms of breast cancer screening: an independent review. *Lancet* 2012;**380**:1778–86. doi:10.1016/S0140-6736(12)61611-0

7 Beral V, Banks E, Bull D, *et al.* Breast cancer and hormone replacement therapy in the Million Women Study. *Lancet* 2003;**362**:419–27. doi:10.1080/13697130400014698

For peer review only - http://bmjopen.bmj.com/site/about/guidelines.xhtml

8 Rossouw JE, Anderson GL, Prentice RL, *et al.* Risks and benefits of estrogen plus progestin in healthy postmenopausal women: principal results From the Women's Health Initiative randomized controlled trial. *JAMA* 2002;**288**:321–33.

9 Lagerlund M, Drake I, Wirfält E, *et al.* Health-related lifestyle factors and mammography screening attendance in a Swedish cohort study. *Eur J Cancer Prev* 2015;**24**:44–50. doi:10.1097/CEJ.0000000000000025

10 Ibrayev Y, Oda K, Fraser GE, *et al.* Utilization of prostate cancer screening according to dietary patterns and other demographic variables. The adventist health study-2. *J Cancer* 2013;**4**:416–26. doi:10.7150/jca.6442

11 Crowe FL, Appleby PN, Travis RC, *et al.* Risk of hospitalization or death from ischemic heart disease among British vegetarians and nonvegetarians: results from the EPIC-Oxford cohort study. *Am J Clin Nutr* 2013;**97**:597–603. doi:10.3945/ajcn.112.044073

12 Appleby PN, Crowe FL, Bradbury KE, *et al.* Mortality in vegetarians and comparable nonvegetarians in the United Kingdom. *Am J Clin Nutr* 2016;**103**:218–30. doi:10.3945/ajcn.115.119461

13 Alewaeters K, Clarys P, Hebbelinck M, *et al.* Cross-sectional analysis of BMI and some lifestyle variables in Flemish vegetarians compared with non-vegetarians. *Ergonomics* 2005;**48**:1433–44. doi:10.1080/00140130500101031

14 Leitzmann C. Vegetarian nutrition: past, present, future 1 – 3. *Am J Clin Nutr* 2014;**100**:496S–502S. doi:10.3945/ajcn.113.071365.496S

15 Townsend P. *Poverty in the United Kingdom: A Survey of Household Resources and Standards of Living*. London, UK: 1979.

For peer review only

For peer review only - http://bmjopen.bmj.com/site/about/guidelines.xhtml

16 Herbert A. Is cervical screening working? A cytopathologist's view from the United Kingdom. *Hum Pathol* 1997;**28**:120–6.

17 National Health Service. NHS Choices: Breast cancer screening. 2015.http://www.nhs.uk/conditions/breast-cancer-screening/Pages/Introduction.aspx (accessed 27 Feb2017).

18 National Health Service. NHS Choices: Cervical screening. 2015.http://www.nhs.uk/Conditions/Cervical-screening-test/Pages/Introduction.aspx#main-navigation (accessed 27 Feb2017).

19 National Health Service. NHS Choices: Prostate Cancer -PSA testing. 2015.http://www.nhs.uk/Conditions/Cancer-of-the-prostate/Pages/Prevention.aspx (accessed 27 Feb2017).

20 Donnelly TT, Al Khater A-H, Al-Bader SB, *et al.* Beliefs and attitudes about breast cancer and screening practices among Arab women living in Qatar: a cross-sectional study. *BMC Womens Health* 2013;**13**:49. doi:10.1186/1472-6874-13-49

21 Nijs HGT, Essink-Bot ML, DeKoning HJ, *et al.* Why do men refuse or attend population-based screening for prostate cancer? *J Public Health Med* 2000;**22**:312–6. doi:10.1093/pubmed/22.3.312

22 Chan CWH, Yang S, Gu C, *et al.* Perception of Cervical Cancer Risk and Screening Behavior: A Literature Review. *Int J Nurs Knowl* 2014;**26**:2–18. doi:10.1111/2047-3095.12028

23 Jensen LF, Mukai TO, Andersen B, *et al.* The association between general practitioners' attitudes towards breast cancer screening and women's screening participation. *BMC Cancer* 2012;**12**:254. doi:10.1186/1471-2407-12-254

For peer review only - http://bmjopen.bmj.com/site/about/guidelines.xhtml

For peer review only

24 Jepson RG, Hewison J, Thompson A, *et al.* Patient perspectives on information and choice in cancer screening: A qualitative study in the UK. *Soc Sci Med* 2007;**65**:890–9. doi:10.1016/j.socscimed.2007.04.009

25 Scholes S, Faulding S, Mindell J. Use of prescribed medicines. Health Survey for England 2013. 2013.

26 Tatham KC, Patel KP. Suitability of common drugs for patients who avoid animal products. *BMJ* 2014;**348**:g401. doi:10.1136/bmj.g401

27 Hayes JH, Barry MJ. Screening for Prostate Cancer With the Prostate-Specific Antigen Test. *JAMA* 2014;**311**:1143. doi:10.1001/jama.2014.2085

28 Perez-Cornago A, Key TJ, Allen NE, *et al.* Prospective investigation of risk factors for prostate cancer in the UK Biobank cohort study. *Br J Cancer* 2017;:1–10. doi:10.1038/bjc.2017.312

For peer review only

Supplementary text 1: List of 36 named medications on the EPIC-Oxford 10 year follow-up questionnaire.

Alendronate, amlodipine, amitriptyline, aspirin, atenolol, atorvastatin, beclomethasone, bendrofluazide, co-codamol/co-dydramol, contraceptive pill, co-proxamol, diclofenac, digoxin, enalapril, etidronate, frusemide, HRT, ibuprofen, insulin, lisinopril, lithium, Losec/Zoton, metformin, nifedipine, paracetamol, paroxetine, prednisolone, propranolol, Prozac, risedronate, salbutamol, simvastatin, sleeping pills, tamoxifen, thyroxine, warfarin

Supplementary text 2: List of 29 named medical conditions asked on the EPIC-Oxford 10 year follow-up questionnaire.

Cancer (type of cancer), blood clot in leg, blood clot in lung or elsewhere, stroke, transient ischaemic attack, angina, heart attack, palpitations/irregular heart beat (cardiac arrhythmia), diabetes, high blood cholesterol, high blood pressure, asthma, emphysema/chronic bronchitis, thyroid problem, cataract in eye, stomach or duodenal ulcer, bowel polyps, diverticular disease, Crohn's disease/ulcerative colitis, coeliac disease, osteoporosis, rheumatoid arthritis, osteoarthritis, depression/anxiety, gallstones, gallbladder removed, epilepsy, multiple sclerosis, enlarged prostate (men only)

For peer review only

STROBE Statement—checklist of items that should be included in reports of observational studies

	Item No	Recommendation
**Title and abstract**	1	(*a*) Indicate the study's design with a commonly used term in the title or the abstract P.1
		(*b*) Provide in the abstract an informative and balanced summary of what was done and what was found P.2
**Introduction**		
Background/rationale	2	Explain the scientific background and rationale for the investigation being reported P.4
Objectives	3	State specific objectives, including any prespecified hypotheses P.5 Lines 71-74
**Methods**		
Study design	4	Present key elements of study design early in the paper P.5 Lines 78, P.7 Lines 127-129
Setting	5	Describe the setting, locations, and relevant dates, including periods of recruitment, exposure, follow-up, and data collection P.5-7 Lines 77-124
Participants	6	(*a*) *Cohort study*—Give the eligibility criteria, and the sources and methods of selection of participants. Describe methods of follow-up<br>*Case-control study*—Give the eligibility criteria, and the sources and methods of case ascertainment and control selection. Give the rationale for the choice of cases and controls<br>*Cross-sectional study*—Give the eligibility criteria, and the sources and methods of selection of participants P.5-6 Lines 80-87
		(*b*) *Cohort study*—For matched studies, give matching criteria and number of exposed and unexposed<br>*Case-control study*—For matched studies, give matching criteria and the number of controls per case
Variables	7	Clearly define all outcomes, exposures, predictors, potential confounders, and effect modifiers. Give diagnostic criteria, if applicable P.6-9 Lines 98-124, 141-165
Data sources/ measurement	8*	For each variable of interest, give sources of data and details of methods of assessment (measurement). Describe comparability of assessment methods if there is more than one group P.5-7 Lines 81-119
Bias	9	Describe any efforts to address potential sources of bias P.7-8 Lines 133-140
Study size	10	Explain how the study size was arrived at P.5 Lines 82-87, P.9 Lines 169-171
Quantitative variables	11	Explain how quantitative variables were handled in the analyses. If applicable, describe which groupings were chosen and why P.8-9 Lines 144-164
Statistical methods	12	(*a*) Describe all statistical methods, including those used to control for confounding P.7-9 Lines 126-165
		(*b*) Describe any methods used to examine subgroups and interactions P.8 Lines 159-160
		(*c*) Explain how missing data were addressed P.8-9 Lines 148, 158, 160-164
		(*d*) *Cohort study*—If applicable, explain how loss to follow-up was addressed<br>*Case-control study*—If applicable, explain how matching of cases and controls was addressed<br>*Cross-sectional study*—If applicable, describe analytical methods taking account of sampling strategy P.7 Lines 127-129

For peer review only

For peer review only - http://bmjopen.bmj.com/site/about/guidelines.xhtml

(*e*) Describe any sensitivity analyses P.8-9 Lines 159-164

Continued on next page

For peer review only

For peer review only - http://bmjopen.bmj.com/site/about/guidelines.xhtml

**Results**		
Participants	13*	(a) Report numbers of individuals at each stage of study—eg numbers potentially eligible, examined for eligibility, confirmed eligible, included in the study, completing follow-up, and analysed P.9 Lines 169-174
		(b) Give reasons for non-participation at each stage P.9 Lines 169-174
		(c) Consider use of a flow diagram
Descriptive data	14*	(a) Give characteristics of study participants (eg demographic, clinical, social) and information on exposures and potential confounders P.9 Lines 175-177, Table 1
		(b) Indicate number of participants with missing data for each variable of interest P.9 Lines 171-174, 180-181
		(c) *Cohort study*—Summarise follow-up time (eg, average and total amount)
Outcome data	15*	*Cohort study*—Report numbers of outcome events or summary measures over time
		Case-control study—Report numbers in each exposure category, or summary measures of exposure
		Cross-sectional study—Report numbers of outcome events or summary measures P.9-10, Table 2-5
Main results	16	(*a*) Give unadjusted estimates and, if applicable, confounder-adjusted estimates and their precision (eg, 95% confidence interval). Make clear which confounders were adjusted for and why they were included P.9-10, Table 2-5
		(*b*) Report category boundaries when continuous variables were categorized
		(*c*) If relevant, consider translating estimates of relative risk into absolute risk for a meaningful time period
Other analyses	17	Report other analyses done—eg analyses of subgroups and interactions, and sensitivity analyses P.10 Lines 201-204
**Discussion**		
Key results	18	Summarise key results with reference to study objectives P.11 Lines 208-217
Limitations	19	Discuss limitations of the study, taking into account sources of potential bias or imprecision. Discuss both direction and magnitude of any potential bias P.15 Lines 317-326
Interpretation	20	Give a cautious overall interpretation of results considering objectives, limitations, multiplicity of analyses, results from similar studies, and other relevant evidence P.12-15
Generalisability	21	Discuss the generalisability (external validity) of the study results P.11-12, P.15 Lines 322-326
**Other information**		
Funding	22	Give the source of funding and the role of the funders for the present study and, if applicable, for the original study on which the present article is based) P.16 Lines 341-343

*Give information separately for cases and controls in case-control studies and, if applicable, for exposed and unexposed groups in cohort and cross-sectional studies.

Note: An Explanation and Elaboration article discusses each checklist item and gives methodological background and published examples of transparent reporting. The STROBE checklist is best used in conjunction with this article (freely available on the Web sites of PLoS Medicine at http://www.plosmedicine.org/, Annals of Internal Medicine at http://www.annals.org/, and Epidemiology at http://www.epidem.com/). Information on the STROBE Initiative is available at www.strobe-statement.org.

For peer review only

For peer review only - http://bmjopen.bmj.com/site/about/guidelines.xhtml

BMJ Open

Cross-sectional analyses of participation in cancer screening and use of hormone replacement therapy and medications in meat eaters and vegetarians: the EPIC-Oxford study.

Journal:	*BMJ Open*
Manuscript ID	bmjopen-2017-018245.R2
Article Type:	Research
Date Submitted by the Author:	20-Nov-2017
Complete List of Authors:	Tong, Tammy Yin Ni; Cancer Epidemiology Unit,<br>Appleby, P; Cancer Epidemiology Unit, University of Oxford<br>Bradbury, Kathryn; Cancer Epidemiology Unit, University of Oxford<br>Key, Timothy; Cancer Epidemiology Unit, University of Oxford
**Primary Subject Heading**:	Epidemiology
Secondary Subject Heading:	Nutrition and metabolism, Oncology
Keywords:	NUTRITION & DIETETICS, ONCOLOGY, HEALTH SERVICES ADMINISTRATION & MANAGEMENT

SCHOLARONE™
Manuscripts

For peer review only

Cross-sectional analyses of participation in cancer screening and use of hormone replacement therapy and medications in meat eaters and vegetarians: the EPIC-Oxford study.

Tammy Y.N. Tong[1], Paul N. Appleby[1], Kathryn E. Bradbury[1], Timothy J. Key[1]

1. Cancer Epidemiology Unit, Nuffield Department of Population Health, University of Oxford, UK

Corresponding author: Tammy Y.N. Tong

Postal address: Cancer Epidemiology Unit, University of Oxford, Richard Doll Building, Old Road Campus, Oxford, OX3 7LF

Tel: +44 (0) 1865 289653

Email: tammy.tong@ceu.ox.ac.uk

Word count: 3657 words

Keywords: vegetarians; cancer screening; hormone replacement therapy; medication use

For peer review only - http://bmjopen.bmj.com/site/about/guidelines.xhtml

ABSTRACT

Objectives: To examine differences in health-related behaviours such as screening or testing for cancer, use of hormone replacement therapy (HRT), and use of other medications in different diet groups.

Design: We studied 31,260 participants across four diet groups (18,155 meat eaters, 5,012 fish eaters, 7,179 vegetarians, 914 vegans) in the UK EPIC-Oxford cohort. Information was collected in 5 (around 2000-2003) or 10 (around 2007) year follow-up questionnaires regarding participation in breast screening, cervical screening, prostate specific antigen (PSA) testing, use of HRT, and use of medications for the past four weeks. Using Poisson regression, we estimated the prevalence ratios (PR) for each behaviour across people of different diet groups, using meat-eaters as the reference group.

Results: Compared with meat-eaters, vegetarian (PR; 95% confidence interval: 0.94; 0.89, 0.98) and vegan (0.82; 0.71, 0.95) women reported lower participation in breast screening, and vegetarian men were less likely to report PSA testing (0.82; 0.71, 0.96). No differences were observed among women for cervical screening. In women, all non-meat eating groups reported lower use of HRT compared with meat-eaters (p heterogeneity<0.0001). Lower reported use of any medication was observed for participants in all non-meat eating groups with no (p<0.0001) or one (p=0.0002) self-reported illness. No heterogeneity was observed across the diet groups for the reported use of specific medication for high blood pressure, high blood cholesterol, asthma, diabetes, and thyroid disease.

Conclusions: Differences in self-reported breast screening, PSA testing, HRT use and overall medication use were observed across the diet groups. Whether such differences contribute to differential long-term disease risks requires further study.

For peer review only - http://bmjopen.bmj.com/site/about/guidelines.xhtml

Strengths and limitations of this study

- This study is the first to simultaneously examine the reported uptake of breast and cervical cancer screening, prostate specific antigen testing, hormone replacement therapy (HRT) use and medication use in different diet groups.
- The study includes a large number of participants recruited from across different regions in the United Kingdom, with a high proportion of fish eaters, vegetarians, and vegans.
- Recall bias is possible because assessment of cancer screening or testing, HRT use and medication use was based on self-report, although there is no indication that such misclassification bias should differ by diet group.
- The study is cross-sectional and we cannot infer causality.

For peer review only

For peer review only - http://bmjopen.bmj.com/site/about/guidelines.xhtml

INTRODUCTION

People of different habitual diet groups have been shown to have different health characteristics. Compared to meat eaters, vegetarians generally have lower BMI, blood pressure, and circulating low density lipoprotein cholesterol levels [1–3], characteristics likely to reduce disease risk. However, evidence on the long-term risk of many non-communicable diseases across people of different diet groups is limited.

For cancer risk, both a United Kingdom (UK) [4] and a United States (US) [5] study reported lower risk of overall cancer incidence with a vegetarian diet. Because health related behaviours, such as participation in cancer screening [6] or use of hormone replacement therapy (HRT) [7,8], may contribute to the observed rates of cancer, the presence of any differences in these behaviours between diet groups in different populations deserve further investigation. Results from a Swedish cohort [9] and a US cohort [10] showed that vegetarians (including vegans and people who ate fish but not meat) had lower odds of attending breast screening and prostate cancer screening respectively, when compared with meat eaters, and vegetarians also had lower use of HRT compared with non-vegetarians [5].

For cardiovascular diseases, vegetarians in EPIC-Oxford have been observed to have lower ischaemic heart disease risk (hospitalization and death combined) [11], but no significant difference in ischaemic heart disease mortality was observed between diet groups in the same population [12]. The reason for this apparent difference between incidence and mortality is unclear. One possible explanation could be the differential use of appropriate medications in the different diet groups, which subsequently influences disease mortality. In a Belgian population for example, vegetarians had lower use of prescription medications compared to non-vegetarians, but similar use of non-prescription drugs [13].

For peer review only - http://bmjopen.bmj.com/site/about/guidelines.xhtml

The increasing popularity and interest in vegetarian diets [14] prompts research on the long-term health of vegetarians and vegans. Because health behaviour such as screening or medication use may ultimately influence disease risk, the understanding of any differences in these behaviours by diet group is crucial for the appropriate appraisal of possible differences in disease risk between diet groups. However, current knowledge on this topic is insufficient, because literature on participation in screening and use of medication across people of different diet groups is scarce. Therefore, the aim of this study was to assess some of these relevant health behaviours, including participation in cancer screening or testing, and use of HRT and other medications among people of different diet groups, in a large population-based cohort in the UK with a high percentage of vegetarians.

METHODS

Study population

The EPIC-Oxford study is a UK based cohort recruited between 1993 and 1999. The study protocol was approved by a Multicentre Research Ethics Committee (Scotland A Research Ethics Committee) and participants gave written informed consent. Details of the recruitment process have been described previously [1]. In brief, a combination of general practitioner (GP) recruitment and postal recruitment was used. The GP recruitment invited men and women aged 35 to 59 years registered with participating GPs and recruited 7,421 participants. The postal recruitment was targeted at vegetarians, vegans, and other people interested in diet and health, by contacting members of The Vegetarian Society, The Vegan Society, and via leaflets enclosed in vegetarian and health food magazines and displayed in health-food shops, and recruited 57,990 participants aged ≥20 years. Altogether, 57,443 participants completed a full recruitment questionnaire which asked about their personal details (including postcode to

For peer review only - http://bmjopen.bmj.com/site/about/guidelines.xhtml

which a Townsend index of area-level deprivation was assigned [15]), habitual diet and other health and lifestyle characteristics, including personal and family medical history, medication use, socio-economic characteristics, smoking and drinking behaviour, and physical activity levels. A follow-up questionnaire was sent to surviving participants approximately 5 years after recruitment (mostly from 2000 to 2003), and a second follow-up questionnaire was mailed approximately 10 years after recruitment (mostly in 2007). In the follow-up questionnaires, updated information was gathered on diet, health and lifestyle, including self-reported current health. Due to the changing research focus over the course of data collection, slight variations existed between questions asked on the 5 and 10 year follow-up questionnaires.

Assessment of diet group

In the recruitment questionnaire and each subsequent follow-up questionnaire, four questions were asked regarding consumption of meat, fish, dairy products, and eggs, in the form of "Do you eat any meat?" or similar for the other three food groups. Responses to these questions were used to assign participants to one of four diet groups at each time point: meat eaters (participants who ate meat, irrespective of whether they ate fish, dairy products or eggs); fish eaters (participants who did not eat meat but did eat fish); vegetarians (participants who did not eat meat or fish, but did eat one or both of dairy products and eggs), and vegans (participants who did not eat meat, fish, dairy products, or eggs).

For peer review only - http://bmjopen.bmj.com/site/about/guidelines.xhtml

Assessment of participation in screening, HRT and medication use

In the follow-up questionnaires, women were asked if they had ever had a breast screening by mammography, cervical screening by the smear test (only on the 5 year follow-up questionnaire), or used HRT, and men were asked if they had ever had a prostate specific antigen (PSA) test (only on the 10 year follow-up questionnaire). On the 10 year follow-up questionnaire, all participants were asked if they had used any medication for most of the last four weeks, with 36 named medications and a free text field for reporting regular use of any medication not on the list; participants were also asked if they had been diagnosed with any of a list of 29 medical conditions, and the year when the condition was first diagnosed. The full list of the 36 medications and 29 medical conditions is given in Supplementary text 1 and 2. The corresponding question on medication use on the 5 year questionnaire was shorter, with 20 named medications and 26 medical conditions.

For assessment of specific medication use, five common medical conditions associated with specific medications were identified: high blood pressure (commonly treated with one or more of amlodipine, enalapril, frusemide, propranolol, atenolol, bendrofluazide, lisinopril and nifedipine), high blood cholesterol (atorvastatin and simvastatin), asthma (beclomethasone and salbutamol), diabetes (insulin and metformin), and thyroid disease (thyroxine).

Statistical analyses

Information on assignment to diet group and assessment of health behaviour from the 10 year follow-up questionnaire was used for our analyses, except for the assessment of participation in cervical screening which was only asked on the 5 year follow-up questionnaire. Participants were excluded from all analysis if they did not answer the relevant questions to

For peer review only - http://bmjopen.bmj.com/site/about/guidelines.xhtml

be assigned to an appropriate diet group (n=28), and in order to ensure that an overlapping population was used for the analyses of all outcomes, they were also excluded if they did not answer the relevant question on medication use (n=407). For the analyses related to participation in breast screening, cervical screening, PSA testing or HRT use, only women or men who answered the relevant question and were in the specified age group at questionnaire completion were included. The age group specifications were as follows: age 50 to 74 years for breast screening, age 25 to 74 years for cervical screening, age 50 to 84 years for PSA testing, and age 50 to 74 years for HRT use. For HRT use, we further restricted the analysis to post-menopausal women, determined by including only participants who answered yes to the question 'Have you been through your menopause?' on the follow-up questionnaire.

For each analysis, we used Poisson regression to estimate prevalence ratios (95% confidence intervals, CI) of cancer screening or testing (breast screening, cervical screening, PSA testing), HRT use, or medication use in different diet groups, using meat eaters as the reference group. For analyses of cancer screening or testing and use of HRT, we adjusted for age at follow-up (<40, 40-44, 45-49, 50-54, 55-59, 60-64, 65-69, 70-74, ≥75 years as appropriate for the age range included in the analysis), region of recruitment (eight geographical regions across the UK), and self-reported current health (excellent, good, fair, poor, unknown). For analyses of any medication use, we adjusted for the cross-stratification of sex and age at follow-up, region of recruitment, self-reported current health, and the number of self-reported illnesses or conditions (0, 1, 2, 3, ≥4). To further assess whether any variation in medication use by diet group varied by health status, we repeated the analyses stratified by the number of self-reported illnesses or conditions using the above categorisation. Subsequently, for each of high blood pressure, high blood cholesterol, asthma, diabetes, and thyroid disease, we estimated the prevalence ratios of taking appropriate medication by diet group among people diagnosed with each condition in turn, adjusting for

For peer review only - http://bmjopen.bmj.com/site/about/guidelines.xhtml

covariates as above and additionally for years since reported diagnosis, calculated as year of follow-up questionnaire completion minus reported year of diagnosis (<2, 2-3, 4-5, 6-9, ≥10 years, unknown).

As sensitivity analyses, we repeated the analyses as follows: using data from the 5 year follow-up questionnaire where available; and further adjusting for smoking status (never, former, current, unknown), alcohol consumption (<1 g/day, 1-7 g/day, 8-15 g/day, ≥16 g/day), Townsend index of area-level deprivation (quartiles and unknown), and education level (no qualifications, basic secondary e.g. O level, higher secondary e.g. A level, degree, unknown). All statistical analyses were performed using Stata release 14.1 (StataCorp), and *P* values < 0.05 were considered statistically significant.

RESULTS

Cohort characteristics

Overall, 57,443 participants in EPIC-Oxford cohort completed a full recruitment questionnaire, of whom 38,043 (66%) completed the 5 year follow-up questionnaire, and 31,695 (55%) completed the 10 year follow-up questionnaire. After excluding participants who did not answer the relevant questions on diet group or on medication use, data for 31,260 participants who completed the 10 year follow-up questionnaire (18,155 meat eaters, 5,012 fish eaters, 7,179 vegetarians, and 914 vegans) were used for most of the analyses. Characteristics of the participants are presented in **Table 1**. Overall, non-meat eaters were younger, more likely to report having excellent health, less likely to report taking medication in the past four weeks, and less likely to have reported any illnesses or conditions.

For peer review only - http://bmjopen.bmj.com/site/about/guidelines.xhtml

Participation in screening and use of HRT and medications

Overall, 14,016 women were included in the analyses for breast screening, 27,781 women for cervical screening, and 4,783 men for PSA testing (**Table 2**). In women, compared with meat eaters, vegetarians (prevalence ratio; 95% CI: 0.94; 0.89, 0.98) and vegans (0.82; 0.71, 0.95), but not fish eaters (0.96; 0.92, 1.01) had lower reported attendance of breast screening, but no significant heterogeneity was observed between the diet groups for reported participation in cervical screening (*P*-heterogeneity=0.37). In men, vegetarians had lower reported uptake of PSA testing (0.82; 0.71, 0.96) than meat eaters, while the difference in uptake appeared lower but did not reach statistical significance in vegans (0.72; 0.50, 1.02), and was not significantly different in fish eaters (0.99; 0.85, 1.07). For HRT use, women who were non-meat eaters reported lower use (fish eaters: 0.80; 0.73, 0.88; vegetarians: 0.74; 0.68, 0.81; vegans: 0.42; 0.30, 0.60) compared with women who were meat eaters (**Table 3**).

Irrespective of the number of self-reported illnesses and conditions, non-meat eaters reported lower use of any medication (fish eaters: 0.92; 0.87, 0.96; vegetarians: 0.93; 0.89, 0.98; vegans 0.71; 0.63, 0.81) compared with meat eaters (**Table 4**). When the analyses were stratified by the number of self-reported illnesses or conditions, non-meat eaters with no (P<0.0001) or one (P=0.0002) illness or condition reported lower medication use compared with meat eaters, but the association was attenuated and no longer statistically significant among participants with two, three, or four or more illnesses or conditions. For medication use specific to several common illnesses and conditions, no significant differences were observed between the diet groups in the reported use of appropriate medications for high blood pressure, high blood cholesterol, asthma, diabetes, or thyroid disease, among participants diagnosed with each of these conditions (**Table 5**). Results were consistent when we repeated the analyses where possible using data from the 5 year follow-up questionnaire,

For peer review only - http://bmjopen.bmj.com/site/about/guidelines.xhtml

or when we further adjusted for smoking, alcohol consumption, Townsend deprivation index, and education level (Supplementary table 1).

DISCUSSION

Summary of results

In this UK population-based cohort with a large proportion of participants from different diet groups, we generally observed lower participation in breast screening and lower HRT use among women who were non-meat eaters (separately categorised as fish eaters, vegetarians, and vegans) compared with women who were meat eaters. Vegetarian men had lower participation in PSA testing compared with meat eating men, but no significant difference was observed for cervical screening in women across the diet groups. For medication use, non-meat eaters were less likely to report taking medications than meat eaters overall, but there were no significant differences in medication use among people reporting two or more illnesses or conditions, or for people reporting taking specific medications for various self-reported conditions.

Comparison with other studies

Few studies have reported on the participation in cancer screening or testing, HRT use or medication use among people of different diet groups, and no study has assessed all these behaviours simultaneously in the same cohort. For breast cancer screening, consistent with our findings, the Swedish Malmö Diet and Cancer Study reported that non-attendance for breast cancer screening was more likely in people who were vegetarians or vegans (odds ratio or OR; 95% confidence interval: 1.49; 1.11, 1.99) [9]. Analyses of data from the Adventist

For peer review only - http://bmjopen.bmj.com/site/about/guidelines.xhtml

Health Study-2 in the United States and Canada showed that all non-meat eaters were less likely to report PSA testing compared with meat eaters (0.79; 0.66, 0.95 for fish eaters; 0.76; 0.67, 0.86 for vegetarians; and OR 0.50; 0.42, 0.60 for vegans) [10], whereas we only observed a lower reported uptake among the vegetarians but not the fish eaters (nor the vegans, perhaps because of limited numbers) compared with meat eaters in EPIC-Oxford. However, given the much higher rates of PSA testing in the Adventist Health Study-2 (73.3% versus 31.5% in EPIC-Oxford), attitudes towards screening are likely to be different in the two populations, and therefore the results might not be directly comparable. Similar to our study, the Adventist Health Study-2 also reported lower ever use of HRT (adjusted for age and race) in pesco-vegetarians (21.0%) and lacto-vegetarians (20.4%), and the lowest use in vegans (16.2%), when compared with non-vegetarians (22.4%) [5].

For medication use, a cross-sectional study in a Belgian population reported lower use of prescribed medications when comparing vegetarians to a reference Belgian population (25.5% versus 47.3%, p<0.001) [13]. While this is consistent with our findings on overall medication use, the study did not assess the use of medications stratified by the number of illnesses, nor did they assess appropriate medication use for specific medical conditions. No studies were found which examined participation of cervical screening among people of different diet groups.

Interpretation of findings and implications

Our findings indicate differences in some health related behaviours between people of different diet groups, although the reasons behind such differences are unclear. For the observed differences in screening rates, possible explanations could be related to different attitudes towards the screening programmes. In the UK since 1988 [6,16], all women aged 50

For peer review only - http://bmjopen.bmj.com/site/about/guidelines.xhtml

to 70 are invited to attend breast cancer screening clinics [17] and all women aged 25 to 64 are invited for cervical screening [18] at regular intervals. On the other hand, there is no national programme for PSA testing, although men over the age of 50 are eligible to arrange for testing via their GP if they wish [19]. In studies which assessed attitudes towards cancer screening or testing, common reasons which affect people's participation in screening include their education level and knowledge of the procedure, recommendation by their doctor, fear of the procedure or the outcome, or their perceived risk of cancer [20–23]. If vegetarians and vegans felt their diets or lifestyles were protective against cancer for example, they might be more likely to forgo cancer screening as a result of lower perceived risk. However, no information was found on whether or how such attitudes may vary by diet group.

In a small focus group study in Scotland which asked participants about their attitudes towards cancer screening (n=31 for cervical screening, n=10 for breast screening), the study participants reported that they felt pressure from health care professionals, family and friends to attend cervical screening but not breast screening, and that they also considered cervical screening to be normative routine behaviour [24]. Such differences in attitudes towards breast screening and cervical screening are of interest, as this may help to explain the differences we observed in participation for breast screening but not cervical screening, if the latter was considered routine behaviour. However, relevant evidence is lacking, and both dietary and non-dietary factors which are associated with attendance for either breast screening or PSA testing deserve further study.

Reasons for the observed lower prevalence of HRT use and medication use among people of different diet groups are also unclear. The prevalence of medication use in meat eaters (56%) in EPIC-Oxford was slightly higher than the UK average of 43% of men and 50% of women aged 16 or above who reported taking at least one prescribed medicine in the last week [25], confirming the relatively low prevalence of medication use in the vegetarians (39%) and

For peer review only - http://bmjopen.bmj.com/site/about/guidelines.xhtml

vegans (28%). However, given the differences in age ranges and possible differences in medications accounted for, strict comparisons cannot be made. Because lower reported use of medications was observed even in people with no (especially) or only one reported illness or condition, better health among non-meat eaters is unlikely to be the only, or a sufficient explanation for the differences. Non-meat eaters may also be reluctant to take medications which are likely to contain animal-derived products [26], or may prefer to use homoeopathic medications [13] or other alternative therapies. Since information on medication use in this study was based on a pre-specified list from the follow-up questionnaire, it was not possible to assess the use of alternative therapies or any other named medications, despite their possible contributions to prevalence of overall medication use.

Differential participation in screening for breast or prostate cancer, use of HRT, and use of medications for people of distinct diet groups may ultimately lead to differences in disease incidence or prognosis due to possible detection bias and differential post diagnosis treatment. For example, breast cancer screening results in higher incidence but reduced mortality from breast cancer among those who are screened [6]. Prostate cancer testing is also linked to increased incidence in those who are tested [27,28]. Therefore, using breast cancer as an example, given the lower rates of breast cancer screening among non-meat eating women both in EPIC-Oxford and in the Swedish Malmö Diet and Cancer Study [9], it is possible that the observed incidence of breast cancer in these diet groups underestimates the true incidence owing to detection bias, but that ultimately these women would be expected to have a somewhat higher mortality from breast cancer. Therefore, future work on assessing breast cancer risk in people of different diet groups should take into account any differences in screening rates between diet groups.

Similarly, it is not clear why there was differential use of HRT in the four diet groups, for example whether it was because non-meat eaters were less likely to have symptoms, or

For peer review only

For peer review only - http://bmjopen.bmj.com/site/about/guidelines.xhtml

because they were less likely to seek treatment when symptoms appear. Regardless of the underlying reason, the observed lower reported use of HRT among non-meat eating women deserves attention, because use of HRT may confound any observed associations between diet group and breast cancer, given that HRT preparations containing oestrogens and progestogens have been shown to increase the risk of breast cancer [7,8].

Overall, our findings showed some differences in health related behaviours between people of different diet groups, thereby highlighting the need to consider such differences when conducting longitudinal analyses in these populations. Future work should also consider possible differences in other health behaviours between diet groups, such as attendance of colorectal screening. Further study is warranted to understand why people of different diet groups have differential participation in breast screening or prostate cancer testing, HRT use, and overall medication use, whether these differences vary by reasons for adhering to each diet group, and whether or how these differences are related to future disease risk.

Strengths and limitations

This study is the first to simultaneously examine participation in cancer screening or testing, HRT use and medication use in different diet groups. A strength of the study is the large sample size recruited from across different regions in the UK. Additionally, information was collected on a range of factors which may also be associated with the behaviours of interest, allowing adjustment for these factors. Of potential limitations, recall bias is possible because assessment of the behaviours of interest (i.e. breast screening, PSA testing, HRT use and overall medication use) as well as existing medical conditions was based on self-report, although there is no indication that such misclassification bias should differ by diet group. The reasons for which people adhered to each diet group were not recorded, although such

For peer review only - http://bmjopen.bmj.com/site/about/guidelines.xhtml

reasons may be relevant to the other health behaviours studied. Because of the relatively small number of vegans in our study sample, the role of chance in explaining the findings relating to this diet group, especially subgroup analyses related to medication use, cannot be ruled out. As with most population cohorts, some degree of self-selection and healthy cohort bias may also be present.

CONCLUSIONS

In this population, we observed differences in breast screening, PSA testing, HRT use and overall medication use between meat eaters, fish eaters, vegetarians and vegans, but no significant differences between diet groups for cervical screening, or medication use in people with two or more illnesses or for specific conditions. The reasons for these differences require further investigation. Nonetheless, such differences may be related to or could confound any differences in observed morbidity or mortality from cancer and other diseases between people of different diet groups, and therefore should be considered in future epidemiological studies.

STATEMENTS

Acknowledgements

We thank all participants in the EPIC-Oxford cohort for their invaluable contribution. The work is supported by the UK Medical Research Council MR/M012190/1 and Cancer Research UK 570/A16491 and C9221/A19170. KEB is supported by the Girdlers' New Zealand Health Research Council Fellowship. TJK is a member of the Vegan Society; the other authors had no conflicts of interest.

For peer review only - http://bmjopen.bmj.com/site/about/guidelines.xhtml

For peer review only

Availability of data and materials

The data access policy for EPIC-Oxford is available via the study website (http://www.epic-oxford.org/data-access-sharing-and-collaboration/).

Author's contributions

TYNT, PNA, and TJK conceived and designed the research question. TYNT and PNA analysed the data. TYNT wrote the first draft of the manuscript, and PNA, KEB and TJK provided input on data analysis and interpretation of results. All authors revised the manuscript critically for important intellectual content, and read and approved the final manuscript.

For peer review only

For peer review only - http://bmjopen.bmj.com/site/about/guidelines.xhtml

Table 1 Characteristics by diet group of participants in the EPIC-Oxford study who completed the second follow-up questionnaire (n=31260) [1].

Characteristic	Meat eaters	Fish eaters	Vegetarians	Vegans	Total
Number of participants (% female)	18155 (78.2)	5012 (81.8)	7179 (76.3)	914 (66.1)	31260 (78.0)
Mean (SD) age at questionnaire completion, years	58.9 (12.5)	53.8 (12.5)	51.6 (12.7)	50.7 (12.3)	56.1 (13.0)
Smoking status[2], n (%)					
Never smoker	10073 (55.7)	2786 (55.6)	4339 (60.5)	547 (59.9)	17745 (56.9)
Former smoker	6927 (38.3)	1961 (39.2)	2460 (34.3)	330 (36.1)	11678 (37.5)
Current smoker	1094 (6.0)	260 (5.2)	367 (5.1)	36 (3.9)	1757 (5.6)
Mean (SD) alcohol consumption, g/d	8.7 (9.3)	8.2 (8.7)	7.6 (8.9)	6.7 (9.2)	8.3 (9.1)
Self-reported current health[2], n (%)					
Excellent	3713 (21.9)	1323 (28.1)	1950 (28.7)	325 (37.2)	7311 (24.9)
Good	9962 (58.8)	2688 (57.0)	3851 (56.6)	446 (51.0)	16947 (57.8)
Fair	2858 (16.9)	612 (13.0)	876 (12.9)	80 (9.2)	4426 (15.1)
Poor	400 (2.4)	92 (2.0)	122 (1.8)	23 (2.6)	637 (2.2)
Townsend deprivation index[2], n (%)					
Richest category	4463 (27.6)	984 (21.8)	1542 (23.7)	153 (18.3)	7141 (25.5)
Poorest category	3438 (21.2)	1207 (26.8)	1732 (26.7)	285 (34.1)	6662 (23.8)
In same diet group at recruitment, n (%)	15908 (87.7)	3057 (61.1)	6373 (89.1)	573 (62.7)	25911 (83.0)
Taking medication in the past 4 weeks, n (%)	10196 (56.2)	2105 (42.0)	2829 (39.4)	255 (27.9)	15385 (49.2)
Number of reported illnesses and conditions, n (%)					
None	4455 (24.5)	1635 (32.6)	2603 (36.3)	344 (37.6)	9037 (28.9)
One	4724 (26.0)	1472 (29.4)	2170 (30.2)	291 (31.8)	8657 (27.7)
Two	3682 (20.3)	906 (18.1)	1261 (17.6)	154 (16.8)	6003 (19.2)
Three	2404 (13.2)	524 (10.5)	630 (8.8)	74 (8.1)	3632 (11.6)
Four or more	2890 (15.9)	475 (9.5)	515 (7.2)	51 (5.6)	3931 (12.6)
Reported high blood pressure[2], n (%)	4397 (29.2)	686 (16.2)	944 (15.2)	85 (10.6)	6112 (23.2)
and taking appropriate medication, n (%)	2573 (58.5)	357 (52.0)	430 (45.6)	40 (47.1)	3400 (55.6)
Reported high blood cholesterol[2], n (%)	3351 (23.1)	561 (13.5)	645 (10.5)	44 (5.5)	4601 (18.0)
and taking appropriate medication, n (%)	1646 (49.1)	209 (37.3)	243 (37.7)	14 (31.8)	2112 (45.9)
Reported asthma[2], n (%)	1885 (13.6)	496 (12.1)	758 (12.4)	88 (11.1)	3227 (12.9)
and taking appropriate medication, n (%)	737 (39.1)	169 (34.1)	246 (32.5)	17 (19.3)	1169 (36.2)
Reported diabetes[2], n (%)	707 (5.2)	75 (1.9)	119 (2.0)	7 (0.9)	908 (3.7)
and taking appropriate medication, n (%)	446 (63.1)	41 (54.7)	84 (70.6)	6 (85.7)	577 (63.5)
Reported thyroid disease[2], n (%)	1545 (11.1)	380 (9.2)	465 (7.6)	56 (7.1)	2446 (9.8)
and taking appropriate medication, n (%)	1191 (77.1)	273 (71.8)	337 (72.5)	37 (66.1)	1838 (75.1)

1. Based on participant characteristics at the time of the second follow-up questionnaire (completed approximately10 years from baseline, around 2007).
2. Unknown for some participants.

For peer review only - http://bmjopen.bmj.com/site/about/guidelines.xhtml

Table 2 Participation in screening by diet group of women and men in the EPIC-Oxford study.

Screening/Diet group	Number answering the relevant question [1]	Number (%) answering in the affirmative [1]	Prevalence ratio (95% CI) [1]
**Breast screening[2]**			
Meat eaters	9239	8813 (95.4)	1.00 (ref)
Fish eaters	2143	1928 (90.0)	0.96 (0.92,1.01)
Vegetarians	2395	2078 (86.8)	0.94 (0.89,0.98)
Vegans	239	182 (76.2)	0.82 (0.71,0.95)
			P-het=0.004
**Cervical screening[3]**			
Meat eaters	15936	15365 (96.4)	1.00 (ref)
Fish eaters	4513	4369 (96.8)	1.00 (0.97,1.03)
Vegetarians	6574	6268 (95.3)	0.98 (0.95,1.01)
Vegans	758	691 (91.2)	0.94 (0.87,1.02)
			P-het=0.37
**Prostate specific antigen testing[4]**			
Meat eaters	3078	1066 (34.6)	1.00 (ref)
Fish eaters	594	181 (30.5)	0.99 (0.85,1.17)
Vegetarians	947	228 (24.1)	0.82 (0.71,0.96)
Vegans	164	33 (20.1)	0.72 (0.50,1.02)
			P-het=0.023

1. Number answering the relevant question and number (%) answering in the affirmative were as observed. Prevalence ratios were adjusted for age at follow-up (<40, 40-44, 45-49, 50-54, 55-59, 60-64, 65-69, 70-74, ≥75 years, as appropriate according to the age range of included participants), region of residence (eight regions), and self-reported current health (excellent, good, fair, poor, unknown).
2. Included women aged 50 to 74 who answered the relevant question on the second (10 year) follow-up questionnaire.
3. Included women aged 25 to 74 who answered the relevant question on the first (5 year) follow-up questionnaire
4. Included men aged 50 to 84 who answered the relevant question on the second (10 year) follow-up questionnaire.

Table 3 Use of hormone replacement therapy by diet group of women in the EPIC-Oxford study.

Diet group	Number answering the relevant question [1]	Number (%) answering in the affirmative [1]	Prevalence ratio (95% CI) [1]
Meat eaters	6911	3098 (44.8)	1.00 (ref)
Fish eaters	1614	541 (33.5)	0.80 (0.73,0.88)
Vegetarians	1778	541 (30.4)	0.74 (0.68,0.81)
Vegans	188	31 (16.5)	0.42 (0.30,0.60)
			P-het<0.0001

1. Number answering the relevant question and number (%) answering in the affirmative were as observed. Prevalence ratios were adjusted for age at follow-up 50-54, 55-59, 60-64, 65-69, 70-74 years), region of residence (eight regions), and self-reported current health (excellent, good, fair, poor, unknown). Included post-menopausal women aged 50 to 74 who answered the relevant question on the second (10 year) follow-up questionnaire.

For peer review only

For peer review only - http://bmjopen.bmj.com/site/about/guidelines.xhtml

Table 4 Medication use by number of self-reported illnesses or conditions and diet group of participants in the EPIC-Oxford study. [1]

Number of self-reported illnesses or conditions / Diet group	Number of participants [2]	Percentage taking any medication [2]	Prevalence ratio (95% CI) [2]
**Any number** [3]			
Meat eaters	18155	56.2	1.00 (ref)
Fish eaters	5012	42.0	0.92 (0.87-0.96)
Vegetarians	7179	39.4	0.93 (0.89-0.98)
Vegans	914	27.9	0.71 (0.63-0.81)
			P-het<0.0001
**None**			
Meat eaters	4455	16.9	1.00 (ref)
Fish eaters	1635	11.9	0.80 (0.68-0.94)
Vegetarians	2603	11.5	0.80 (0.70-0.92)
Vegans	344	6.1	0.47 (0.30-0.72)
			P-het<0.0001
**One**			
Meat eaters	4724	48.9	1.00 (ref)
Fish eaters	1472	39.1	0.87 (0.80-0.96)
Vegetarians	2170	40.5	0.91 (0.84-0.99)
Vegans	291	29.2	0.69 (0.55-0.85)
			P-het=0.0002
**Two**			
Meat eaters	3682	66.9	1.00 (ref)
Fish eaters	906	58.8	0.94 (0.86-1.04)
Vegetarians	1261	58.1	0.97 (0.89-1.06)
Vegans	154	42.2	0.74 (0.58-0.95)
			P-het=0.082
**Three**			
Meat eaters	2404	82.6	1.00 (ref)
Fish eaters	524	74.0	0.94 (0.84-1.05)
Vegetarians	630	73.0	0.94 (0.84-1.04)
Vegans	74	59.5	0.78 (0.57-1.05)
			P-het=0.22
**Four or more**			
Meat eaters	2890	93.0	1.00 (ref)
Fish eaters	475	86.9	0.96 (0.86-1.06)
Vegetarians	515	88.9	0.98 (0.89-1.09)
Vegans	51	78.4	0.87 (0.63-1.19)
			P-het=0.70

1. Refers to medication use for most of the past four weeks on the second (10 year) follow-up questionnaire, excluding HRT and contraceptive pills.
2. Number of participants and percentage taking any medication were as observed. Prevalence ratios were adjusted for the cross-classification of sex and age at follow-up (<40, 40-44, 45-49, 50-54, 55-59, 60-64, 65-69, 70-74, ≥75 years), region of residence (eight regions), and self-reported current health (excellent, good, fair, poor, unknown).
3. Prevalence ratios for this category were further adjusted for the number of self-reported illnesses or conditions (0, 1, 2, 3, ≥4).

For peer review only

Table 5 Medication use for specific conditions by diet group of participants in the EPIC-Oxford study.[1]

Condition/ Diet group	Number reporting the condition (mean years since reported diagnosis) [2]	Number (%) taking appropriate medication [2]	Prevalence ratio (95% CI) [2]
**High blood pressure**[3]			
Meat eaters	4397 (9.8)	2573 (58.5)	1.00 (ref)
Fish eaters	686 (9.3)	357 (52.0)	0.97 (0.86-1.08)
Vegetarians	944 (9.0)	430 (45.6)	0.91 (0.82-1.01)
Vegans	85 (9.0)	40 (47.1)	0.92 (0.67-1.26)
			P-het=0.37
**High blood cholesterol**[4]			
Meat eaters	3351 (6.3)	1646 (49.1)	1.00 (ref)
Fish eaters	561 (5.3)	209 (37.3)	0.88 (0.76-1.01)
Vegetarians	645 (5.5)	243 (37.7)	0.94 (0.81-1.08)
Vegans	44 (7.1)	14 (31.8)	0.74 (0.44-1.26)
			P-het=0.20
**Asthma**[5]			
Meat eaters	1885 (25.3)	737 (39.1)	1.00 (ref)
Fish eaters	496 (23.2)	169 (34.1)	0.98 (0.82-1.17)
Vegetarians	758 (23.4)	246 (32.5)	0.97 (0.84-1.14)
Vegans	88 (27.9)	17 (19.3)	0.67 (0.41-1.09)
			P-het=0.45
**Diabetes**[6]			
Meat eaters	707 (10.0)	446 (63.1)	1.00 (ref)
Fish eaters	75 (14.8)	41 (54.7)	0.78 (0.56-1.08)
Vegetarians	119 (10.6)	84 (70.6)	1.05 (0.81-1.35)
Vegans	7 (13.2)	6 (85.7)	1.07 (0.45-2.51)
			P-het=0.46
**Thyroid disease**[7]			
Meat eaters	1545 (13.2)	1191 (77.1)	1.00 (ref)
Fish eaters	380 (11.6)	273 (71.8)	0.95 (0.83-1.09)
Vegetarians	465 (11.2)	337 (72.5)	0.97 (0.85-1.10)
Vegans	56 (11.8)	37 (66.1)	0.88 (0.63-1.22)
			P-het=0.78

1. Refers to medication use for most the past four weeks specific to the condition described, among participants who reported diagnosis for the condition on the second (10 year) follow-up questionnaire.
2. Number reporting the condition (mean years since reported diagnosis) and number (%) taking appropriate medication were as observed. Prevalence ratios were adjusted for the cross-classification of sex and age at follow-up (<40, 40-44, 45-49, 50-54, 55-59, 60-64, 65-69, 70-74, ≥75 years), region of residence (eight regions), self-reported current health (excellent, good, fair, poor, unknown), years since reported diagnosis (calculated as year of follow-up questionnaire completion minus reported year of diagnosis; <2, 2-3, 4-5, 6-9, ≥10 years, unknown), and number of self-reported illnesses or conditions (1, 2, 3, ≥4).
3. Reported use of at least one of amlodipine, enalapril, frusemide, propranolol, atenolol, bendrofluazide, lisinopril and nifedipine.
4. Reported use of at least one of atorvastatin and simvastatin.
5. Reported use of at least one of beclomethasone and salbutamol.
6. Reported use of at least one of insulin and metformin.
7. Reported use of thyroxine.

For peer review only - http://bmjopen.bmj.com/site/about/guidelines.xhtml

REFERENCES

1 Davey GK, Spencer EA, Appleby PN, *et al.* EPIC-Oxford: lifestyle characteristics and nutrient intakes in a cohort of 33 883 meat-eaters and 31 546 non meat-eaters in the UK. *Public Health Nutr* 2003;**6**:259–68. doi:10.1079/PHN2002430

2 Appleby PN, Davey GK, Key TJ. Hypertension and blood pressure among meat eaters, fish eaters, vegetarians and vegans in EPIC-Oxford. *Public Health Nutr* 2002;**5**:645–54. doi:10.1079/PHN2002332

3 Wang F, Zheng J, Yang B, *et al.* Effects of Vegetarian Diets on Blood Lipids: A Systematic Review and Meta-Analysis of Randomized Controlled Trials. *J Am Heart Assoc* 2015;**4**:e002408. doi:10.1161/JAHA.115.002408

4 Key TJ, Appleby PN, Crowe FL, *et al.* Cancer in British vegetarians: Updated analyses of 4998 incident cancers in a cohort of 32,491 meat eaters, 8612 fish eaters, 18,298 vegetarians, and 2246 vegans. *Am J Clin Nutr* 2014;**100**:378–85. doi:10.3945/ajcn.113.071266

5 Tantamango-Bartley Y, Jaceldo-Siegl K, Fan J, *et al.* Vegetarian Diets and the Incidence of Cancer in a Low-risk Population. *Cancer Epidemiol Biomarkers Prev* 2013;**22**:286–94. doi:10.1158/1055-9965.EPI-12-1060

6 Independent UK Panel on Breast Cancer Screening. The benefits and harms of breast cancer screening: an independent review. *Lancet* 2012;**380**:1778–86. doi:10.1016/S0140-6736(12)61611-0

7 Beral V, Banks E, Bull D, *et al.* Breast cancer and hormone replacement therapy in the Million Women Study. *Lancet* 2003;**362**:419–27. doi:10.1080/13697130400014698

For peer review only - http://bmjopen.bmj.com/site/about/guidelines.xhtml

8 Rossouw JE, Anderson GL, Prentice RL, *et al.* Risks and benefits of estrogen plus progestin in healthy postmenopausal women: principal results From the Women's Health Initiative randomized controlled trial. *JAMA* 2002;**288**:321–33.

9 Lagerlund M, Drake I, Wirfält E, *et al.* Health-related lifestyle factors and mammography screening attendance in a Swedish cohort study. *Eur J Cancer Prev* 2015;**24**:44–50. doi:10.1097/CEJ.0000000000000025

10 Ibrayev Y, Oda K, Fraser GE, *et al.* Utilization of prostate cancer screening according to dietary patterns and other demographic variables. The adventist health study-2. *J Cancer* 2013;**4**:416–26. doi:10.7150/jca.6442

11 Crowe FL, Appleby PN, Travis RC, *et al.* Risk of hospitalization or death from ischemic heart disease among British vegetarians and nonvegetarians: results from the EPIC-Oxford cohort study. *Am J Clin Nutr* 2013;**97**:597–603. doi:10.3945/ajcn.112.044073

12 Appleby PN, Crowe FL, Bradbury KE, *et al.* Mortality in vegetarians and comparable nonvegetarians in the United Kingdom. *Am J Clin Nutr* 2016;**103**:218–30. doi:10.3945/ajcn.115.119461

13 Alewaeters K, Clarys P, Hebbelinck M, *et al.* Cross-sectional analysis of BMI and some lifestyle variables in Flemish vegetarians compared with non-vegetarians. *Ergonomics* 2005;**48**:1433–44. doi:10.1080/00140130500101031

14 Leitzmann C. Vegetarian nutrition: past, present, future 1 – 3. *Am J Clin Nutr* 2014;**100**:496S–502S. doi:10.3945/ajcn.113.071365.496S

15 Townsend P. *Poverty in the United Kingdom: A Survey of Household Resources and Standards of Living*. London, UK: 1979.

For peer review only - http://bmjopen.bmj.com/site/about/guidelines.xhtml

16 Herbert A. Is cervical screening working? A cytopathologist's view from the United Kingdom. *Hum Pathol* 1997;**28**:120–6.

17 National Health Service. NHS Choices: Breast cancer screening. 2015.http://www.nhs.uk/conditions/breast-cancer-screening/Pages/Introduction.aspx (accessed 27 Feb2017).

18 National Health Service. NHS Choices: Cervical screening. 2015.http://www.nhs.uk/Conditions/Cervical-screening-test/Pages/Introduction.aspx#main-navigation (accessed 27 Feb2017).

19 National Health Service. NHS Choices: Prostate Cancer -PSA testing. 2015.http://www.nhs.uk/Conditions/Cancer-of-the-prostate/Pages/Prevention.aspx (accessed 27 Feb2017).

20 Donnelly TT, Al Khater A-H, Al-Bader SB, *et al.* Beliefs and attitudes about breast cancer and screening practices among Arab women living in Qatar: a cross-sectional study. *BMC Womens Health* 2013;**13**:49. doi:10.1186/1472-6874-13-49

21 Nijs HGT, Essink-Bot ML, DeKoning HJ, *et al.* Why do men refuse or attend population-based screening for prostate cancer? *J Public Health Med* 2000;**22**:312–6. doi:10.1093/pubmed/22.3.312

22 Chan CWH, Yang S, Gu C, *et al.* Perception of Cervical Cancer Risk and Screening Behavior: A Literature Review. *Int J Nurs Knowl* 2014;**26**:2–18. doi:10.1111/2047-3095.12028

23 Jensen LF, Mukai TO, Andersen B, *et al.* The association between general practitioners' attitudes towards breast cancer screening and women's screening participation. *BMC Cancer* 2012;**12**:254. doi:10.1186/1471-2407-12-254

For peer review only - http://bmjopen.bmj.com/site/about/guidelines.xhtml

24 Jepson RG, Hewison J, Thompson A, *et al.* Patient perspectives on information and choice in cancer screening: A qualitative study in the UK. *Soc Sci Med* 2007;**65**:890–9. doi:10.1016/j.socscimed.2007.04.009

25 Scholes S, Faulding S, Mindell J. Use of prescribed medicines. Health Survey for England 2013. 2013.

26 Tatham KC, Patel KP. Suitability of common drugs for patients who avoid animal products. *BMJ* 2014;**348**:g401. doi:10.1136/bmj.g401

27 Hayes JH, Barry MJ. Screening for Prostate Cancer With the Prostate-Specific Antigen Test. *JAMA* 2014;**311**:1143. doi:10.1001/jama.2014.2085

28 Perez-Cornago A, Key TJ, Allen NE, *et al.* Prospective investigation of risk factors for prostate cancer in the UK Biobank cohort study. *Br J Cancer* 2017;:1–10. doi:10.1038/bjc.2017.312

For peer review only

Supplementary text 1: List of 36 named medications on the EPIC-Oxford 10 year follow-up questionnaire.

Alendronate, amlodipine, amitriptyline, aspirin, atenolol, atorvastatin, beclomethasone, bendrofluazide, co-codamol/co-dydramol, contraceptive pill, co-proxamol, diclofenac, digoxin, enalapril, etidronate, frusemide, HRT, ibuprofen, insulin, lisinopril, lithium, Losec/Zoton, metformin, nifedipine, paracetamol, paroxetine, prednisolone, propranolol, Prozac, risedronate, salbutamol, simvastatin, sleeping pills, tamoxifen, thyroxine, warfarin

Supplementary text 2: List of 29 named medical conditions asked on the EPIC-Oxford 10 year follow-up questionnaire.

Cancer (type of cancer), blood clot in leg, blood clot in lung or elsewhere, stroke, transient ischaemic attack, angina, heart attack, palpitations/irregular heart beat (cardiac arrhythmia), diabetes, high blood cholesterol, high blood pressure, asthma, emphysema/chronic bronchitis, thyroid problem, cataract in eye, stomach or duodenal ulcer, bowel polyps, diverticular disease, Crohn’s disease/ulcerative colitis, coeliac disease, osteoporosis, rheumatoid arthritis, osteoarthritis, depression/anxiety, gallstones, gallbladder removed, epilepsy, multiple sclerosis, enlarged prostate (men only)

For peer review only

Supplementary table 1: Sensitivity analyses using data from the 5 year follow-up questionnaire or with further adjustment for possible confounders.

Sensitivity analyses / Health behaviour of interest	Diet group, prevalence ratio (95% CI)			
	**Meat eaters**	**Fish eaters**	**Vegetarians**	**Vegans**
**Using data from the 5 year follow-up questionnaire**				
Breast screening[1]	1.00 (ref)	0.95 (0.90-1.00)	0.94 (0.89-0.99)	0.77 (0.67-0.89)
Hormone replacement therapy use[1]	1.00 (ref)	0.82 (0.76-0.89)	0.73 (0.67-0.80)	0.52 (0.40-0.68)
**Further adjustment for confounders**				
Breast screening[2]	1.00 (ref)	0.97 (0.92-1.02)	0.94 (0.89-0.99)	0.83 (0.71-0.96)
Cervical screening[2]	1.00 (ref)	1.00 (0.97-1.03)	0.99 (0.96-1.02)	0.95 (0.99-1.02)
Prostate specific antigen testing[2]	1.00 (ref)	1.00 (0.85-1.17)	0.83 (0.72-0.97)	0.76 (0.53-1.08)
Hormone replacement therapy use[2]	1.00 (ref)	0.81 (0.73-0.88)	0.76 (0.69-0.84)	0.44 (0.31-0.63)
Any medication use[3]	1.00 (ref)	0.92 (0.88-0.97)	0.94 (0.90-0.98)	0.71 (0.63-0.81)

1. Adjusted for age at follow-up (<40, 40-44, 45-49, 50-54, 55-59, 60-64, 65-69, 70-74, ≥75 years, as appropriate according to the age range of included participants), region of residence (eight regions), and self-reported current health (excellent, good, fair, poor, unknown).
2. Adjusted for age at follow-up (<40, 40-44, 45-49, 50-54, 55-59, 60-64, 65-69, 70-74, ≥75 years, as appropriate according to the age range of included participants), region of residence (eight regions), self-reported current health (excellent, good, fair, poor, unknown), smoking status (never, former, current, unknown), alcohol consumption (<1 g/day, 1-7 g/day, 8-15 g/day, ≥16 g/day), Townsend index of area-level deprivation (quartiles and unknown), and education level (no qualifications, basic secondary e.g. O level, higher secondary e.g. A level, degree, unknown).
3. Adjusted for the cross-classification of sex and age at follow-up (<40, 40-44, 45-49, 50-54, 55-59, 60-64, 65-69, 70-74, ≥75 years), region of residence (eight regions), self-reported current health (excellent, good, fair, poor, unknown), number of self-reported illnesses or conditions (0, 1, 2, 3, ≥4), smoking status (never, former, current, unknown), alcohol consumption (<1 g/day, 1-7 g/day, 8-15 g/day, ≥16 g/day), Townsend index of area-level deprivation (quartiles and unknown), and education level (no qualifications, basic secondary e.g. O level, higher secondary e.g. A level, degree, unknown).

For peer review only

STROBE Statement—checklist of items that should be included in reports of observational studies

	Item No	Recommendation
**Title and abstract**	1	(*a*) Indicate the study's design with a commonly used term in the title or the abstract P.1
		(*b*) Provide in the abstract an informative and balanced summary of what was done and what was found P.2
**Introduction**		
Background/rationale	2	Explain the scientific background and rationale for the investigation being reported P.4
Objectives	3	State specific objectives, including any prespecified hypotheses P.5 Lines 71-74
**Methods**		
Study design	4	Present key elements of study design early in the paper P.5 Lines 78, P.7 Lines 127-129
Setting	5	Describe the setting, locations, and relevant dates, including periods of recruitment, exposure, follow-up, and data collection P.5-7 Lines 77-124
Participants	6	(*a*) *Cohort study*—Give the eligibility criteria, and the sources and methods of selection of participants. Describe methods of follow-up *Case-control study*—Give the eligibility criteria, and the sources and methods of case ascertainment and control selection. Give the rationale for the choice of cases and controls *Cross-sectional study*—Give the eligibility criteria, and the sources and methods of selection of participants P.5-6 Lines 80-87
		(*b*) *Cohort study*—For matched studies, give matching criteria and number of exposed and unexposed *Case-control study*—For matched studies, give matching criteria and the number of controls per case
Variables	7	Clearly define all outcomes, exposures, predictors, potential confounders, and effect modifiers. Give diagnostic criteria, if applicable P.6-9 Lines 98-124, 141-165
Data sources/ measurement	8*	For each variable of interest, give sources of data and details of methods of assessment (measurement). Describe comparability of assessment methods if there is more than one group P.5-7 Lines 81-119
Bias	9	Describe any efforts to address potential sources of bias P.7-8 Lines 133-140
Study size	10	Explain how the study size was arrived at P.5 Lines 82-87, P.9 Lines 169-171
Quantitative variables	11	Explain how quantitative variables were handled in the analyses. If applicable, describe which groupings were chosen and why P.8-9 Lines 144-164
Statistical methods	12	(*a*) Describe all statistical methods, including those used to control for confounding P.7-9 Lines 126-165
		(*b*) Describe any methods used to examine subgroups and interactions P.8 Lines 159-160
		(*c*) Explain how missing data were addressed P.8-9 Lines 148, 158, 160-164
		(*d*) *Cohort study*—If applicable, explain how loss to follow-up was addressed *Case-control study*—If applicable, explain how matching of cases and controls was addressed *Cross-sectional study*—If applicable, describe analytical methods taking account of sampling strategy P.7 Lines 127-129

For peer review only - http://bmjopen.bmj.com/site/about/guidelines.xhtml

(*e*) Describe any sensitivity analyses P.8-9 Lines 159-164

Continued on next page

For peer review only

**Results**		
Participants	13*	(a) Report numbers of individuals at each stage of study—eg numbers potentially eligible, examined for eligibility, confirmed eligible, included in the study, completing follow-up, and analysed P.9 Lines 169-174
		(b) Give reasons for non-participation at each stage P.9 Lines 169-174
		(c) Consider use of a flow diagram
Descriptive data	14*	(a) Give characteristics of study participants (eg demographic, clinical, social) and information on exposures and potential confounders P.9 Lines 175-177, Table 1
		(b) Indicate number of participants with missing data for each variable of interest P.9 Lines 171-174, 180-181
		(c) *Cohort study*—Summarise follow-up time (eg, average and total amount)
Outcome data	15*	*Cohort study*—Report numbers of outcome events or summary measures over time
		Case-control study—Report numbers in each exposure category, or summary measures of exposure
		Cross-sectional study—Report numbers of outcome events or summary measures P.9-10, Table 2-5
Main results	16	(*a*) Give unadjusted estimates and, if applicable, confounder-adjusted estimates and their precision (eg, 95% confidence interval). Make clear which confounders were adjusted for and why they were included P.9-10, Table 2-5
		(*b*) Report category boundaries when continuous variables were categorized
		(*c*) If relevant, consider translating estimates of relative risk into absolute risk for a meaningful time period
Other analyses	17	Report other analyses done—eg analyses of subgroups and interactions, and sensitivity analyses P.10 Lines 201-204
**Discussion**		
Key results	18	Summarise key results with reference to study objectives P.11 Lines 208-217
Limitations	19	Discuss limitations of the study, taking into account sources of potential bias or imprecision. Discuss both direction and magnitude of any potential bias P.15 Lines 317-326
Interpretation	20	Give a cautious overall interpretation of results considering objectives, limitations, multiplicity of analyses, results from similar studies, and other relevant evidence P.12-15
Generalisability	21	Discuss the generalisability (external validity) of the study results P.11-12, P.15 Lines 322-326
**Other information**		
Funding	22	Give the source of funding and the role of the funders for the present study and, if applicable, for the original study on which the present article is based) P.16 Lines 341-343

*Give information separately for cases and controls in case-control studies and, if applicable, for exposed and unexposed groups in cohort and cross-sectional studies.

Note: An Explanation and Elaboration article discusses each checklist item and gives methodological background and published examples of transparent reporting. The STROBE checklist is best used in conjunction with this article (freely available on the Web sites of PLoS Medicine at http://www.plosmedicine.org/, Annals of Internal Medicine at http://www.annals.org/, and Epidemiology at http://www.epidem.com/). Information on the STROBE Initiative is available at www.strobe-statement.org.

For peer review only
